# Distinct phases of adult microglia proliferation: a *Myc*-mediated early phase and a *Tnfaip3*-mediated late phase

**DOI:** 10.1038/s41421-022-00377-3

**Published:** 2022-04-12

**Authors:** Wulin Tan, Po-Yi Paul Su, Jacqueline Leff, Xiang Gao, Jiao Chen, Andrew K. Guan, Gokul Kalyanasundaram, Averil Ma, Zhonghui Guan

**Affiliations:** 1 Department of Anesthesia and Perioperative Care, University of California San Francisco, San Francisco, CA USA; 2 Department of Anesthesiology, the First Affiliated Hospital, Sun Yat-Sen University, Guangzhou, China; 3 Department of Pharmacy, the First Affiliated Hospital, Sun Yat-Sen University, Guangzhou, China; 4 Department of Medicine, University of California San Francisco, San Francisco, CA USA

**Keywords:** Mechanisms of disease, Transcriptomics

## Abstract

Microgliosis is a hallmark of many neurological diseases, including Alzheimer’s disease, stroke, seizure, traumatic brain and spinal cord injuries, and peripheral and optic nerve injuries. Recent studies have shown that the newly self-renewed microglia have specific neurological functions. However, the mechanism of adult microglia proliferation remains largely unclear. Here, with single-cell RNA sequencing, flow cytometry, and immunohistochemistry, we demonstrate that the sciatic nerve injury induced two distinct phases of microglia proliferation in mouse spinal cord, each with different gene expression profiles. We demonstrate that the transcription factor *Myc* was transiently upregulated in spinal cord microglia after nerve injury to mediate an early phase microglia proliferation. On the other hand, we reveal that the tumor-necrosis factor alpha-induced protein 3 (*Tnfaip3*) was downregulated to mediate the *Myc*-independent late-phase microglia proliferation. We show that cyclin dependent kinase 1, a kinase with important function in the M phase of the cell cycle, was involved only in the early phase. We reveal that although the early phase was neither necessary nor sufficient for the late phase proliferation, the late-phase suppressed the early phase microglia proliferation in the spinal cord. Finally, we demonstrate that the termination of spinal cord microglia proliferation required both *Myc* and *Tnfaip3* to resume their baseline expression. Thus, we have delineated an interactive signaling network in the proliferation of differentiated microglia.

## Introduction

Unlike other cells in central nervous-system (CNS) parenchyma, microglia originate from hematopoietic progenitors in yolk sac and migrate into CNS in early embryonic development^1,2^. Being the tissue macrophages in CNS, adult microglia are critical in keeping normal physiological function and in responding to pathological damages in CNS^3^. One character of adult microglia is that they can proliferate at homeostatic physiological condition and upon pathological stimulations^4–9^. Compared with embryonic microglia, which are highly proliferative^1^, adult microglia have a low rate of baseline proliferation to maintain their homeostatic population^5–7^.

In fact, microglia proliferation occurs in numerous neurological disorders, including Alzheimer’s disease^5,10^, Huntington’s disease^11^, amyotrophic lateral sclerosis (ALS)^12^, stroke^13^, traumatic brain and spinal cord injuries^14,15^, multiple sclerosis^16,17^, seizure^18^, Parkinson’s disease^19^, peripheral and optic nerve injuries^7,9,20^, neuropathic pain^9,21^, infection^22,23^, substance abuse^24^, irradiation^25^, and mental disorders^26^. In Alzheimer’s disease for example, increased microglia proliferation has been observed in both clinical and preclinical nervous system^10^; and the long-term in vivo single-cell imaging study suggests that proliferation of resident microglia is responsible for the increase of microglial numbers around amyloid deposits^5^. Similarly, the expansion of microglia population after peripheral or optic nerve injuries is not associated with infiltration of circulating monocytes^20,27,28^, indicating that microglia proliferation, rather than monocyte infiltration, is responsible for microglia population expansion after nerve injuries. Indeed, with a multicolor fluorescence fate-mapping system, microglia proliferation has been observed in facial nucleus after facial nerve injury^7^. In addition, several parabiotic studies suggest that there is little contribution of circulating monocytes to the expansion of microglia population in the mouse models of experimental autoimmune encephalitis (EAE)^16^, ALS^28^, and stroke^13^. These observations suggest that proliferation of adult microglia is a hallmark of many neurological diseases.

More importantly, adult microglia proliferation contributes to the development of neurological diseases. In a mouse neuropathic pain model, inhibition of microglia proliferation significantly reduces the development of neuropathic pain behavior^21^. Similarly, inhibition of microglia proliferation prevents the behavioral deficits and synaptic degeneration in a mouse model of Alzheimer’s disease^10^. Inhibition of microglia proliferation also prevents neuronal degeneration in prion disease^23^ and attenuates neuronal death in seizure^18^. On the other hand, increased microglia proliferation reduces lesion size and enhances functional recovery after spinal cord injury^14^. In fact, the newborn microglia generated from microglial proliferation have unique transcriptional profile, and they stimulate adult neurogenesis to facilitate the functional recovery after traumatic brain injury^29^. Thus, adult microglia proliferation not only is a hallmark but also has critical functions in the development of many neurological diseases.

Here we report that *Myc* and *Tnfaip3* regulate the early phase and the late phase of microglia proliferation, respectively. We have delineated the interaction between these two pathways, and we have explored the mechanism of the termination of microglia proliferation.

## Results

### Microglia single-cell RNA sequencing (scRNA-Seq)

To investigate the mechanism of microglia proliferation, we applied a mouse sciatic nerve injury model, in which we and others have shown that the nerve injury stimulates microglia proliferation in the corresponding lumbar spinal cord^9,21,27,30^. As microglia are distinguished by *Cx3cr1* expression^1,31^, with young adult *Cx3cr1-creER-yfp/+* heterozygous mice^32^, we sorted YFP (+) cells by fluorescence-activated cell sorting (FACS) from the parenchyma of lumbar spinal cords at different timepoints after nerve injury for 10× Genomic nanoliter droplet-based scRNA-Seq^33^.

We analyzed our scRNA-Seq data with 10× Geneomic Loupe Cell Browser and found that virtually all FACS YFP (+) cells in the mouse lumbar spinal cord are microglia, because 99.97% of sequenced cells expressed *Hexb* (Supplementary Fig. S1), a gene specifically expressed in microglia^34^. Lumbar cord *Cx3cr1*-YFP (+) cells also expressed other microglia signature genes^34^ such as *P2ry12* (99.7% of all cells), *Tmem119* (99.7% of all cells), *Csf1r* (99.7% of all cells), and *Fcrls* (98.3% of all cells). Microglia are distinguished traditionally via flow cytometry as CD11b^high^CD45^intermediate^ markers; we analyzed the microglia from lumbar spinal cord of wild-type animals using flow cytometry (Supplementary Fig. S2) and found that 99% of cells labeled by CD11b^high^CD45^intermediate^ co-expressed CX3CR1, and 98.8% of CX3CR1 (+) cells were CD11b^high^CD45^intermediate^ (Supplementary Fig. S3).

As circulating monocytes also express *Cx3cr1*, along with monocyte-specific gene *Ccr2*^35^, we analyzed our scRNA-Seq of *Cx3cr1*-YFP (+) cells and found minimal amount of *Ccr2*-expressing monocyte in lumbar cord in naive animals, or in animals 1 or 2 days after nerve injury (Supplementary Fig. S1), consistent with the previous report that there is minimal monocyte infiltration in the spinal cord after nerve injury^27^, and that proliferation of existing microglia is the source of microglia expansion after nerve injury^9,21,27,30^. We also found minimal *Cd163* or *Mrc1* expressing perivascular macrophages^31^ in mouse lumbar cord (Supplementary Fig. S1).

**Fig. 1:**
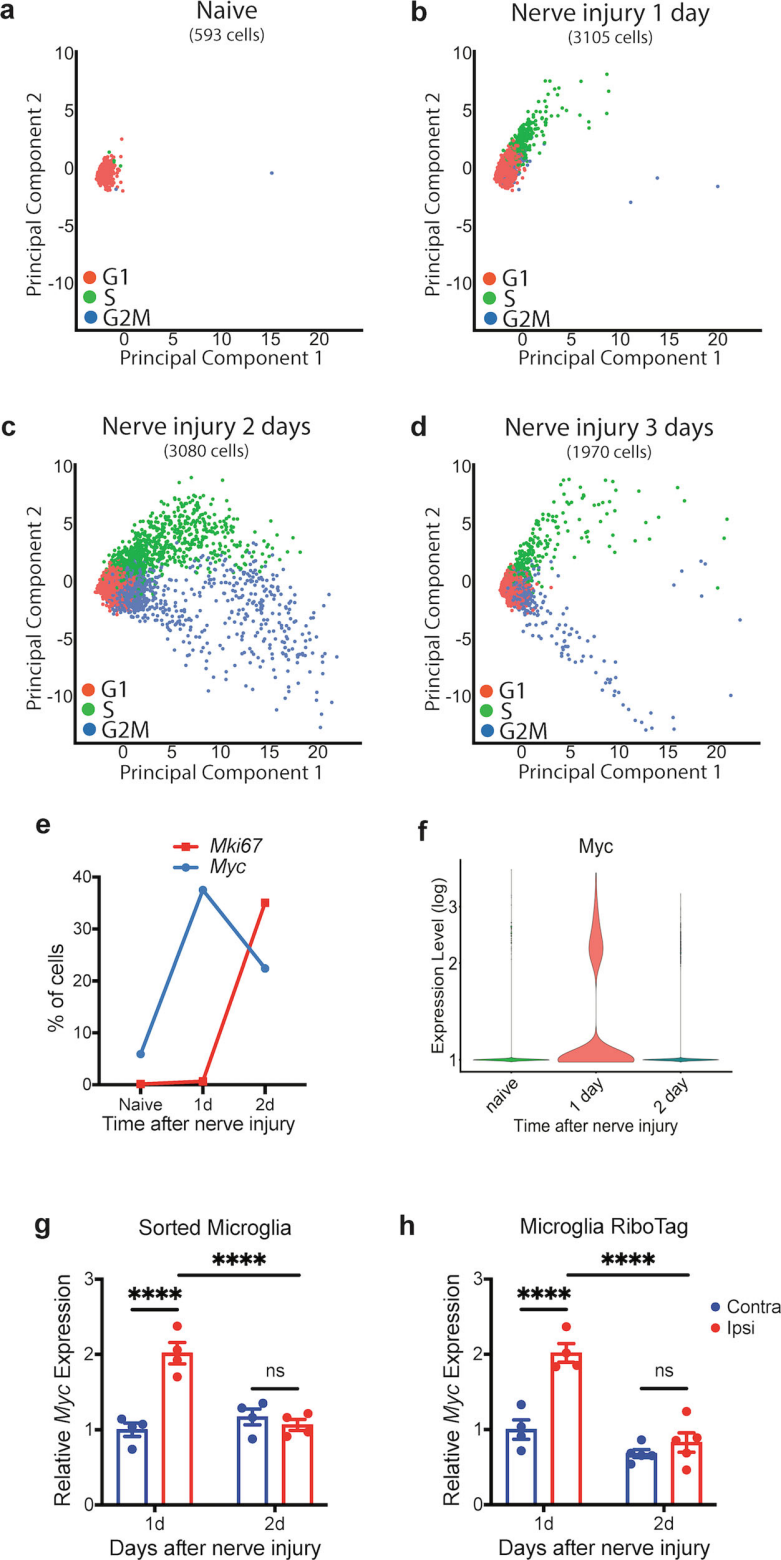
scRNA-Seq of lumbar spinal cord microglia. **a**–**d** scRNA-Seq PCA scatter plots of lumbar spinal cord YFP (+) cells of adult *Cx3cr1-creER-yfp /+; +/+* heterozygous animals at different timepoints after sciatic nerve injury. The vast majority of microglia in naive animal were in G1 phase of the cell cycle (**a**), a substantial portion of microglia 1 day after nerve injury were in S phase of the cell cycle (**b**), and many microglia were in G2/M phase of the cell cycle 2 days (**c**) and 3 days (**d**) after nerve injury. Note that the S-phase cells appeared one day (**b**) before the appearance of G2/M-phase cells (**c**), and the distribution patterns of S phase cells were different between 1 day (**b**) and 2 days (**c**) after nerve injury. The distribution patterns of G2/M phase cells were also different between 2 days (**c**) and 3 days (**d**) after nerve injury. **e** Proportion of microglia cells expressing *Myc* or *Mki67* from scRNA-Seq analysis. *Myc* was expressed in a higher proportion of microglia 1 day after nerve injury, and *Mki67* was expressed in a higher proportion of microglia 2 days after nerve injury. **f** Violin plot of scRNA-Seq showing that a cluster of microglia transiently increased *Myc* expression on day 1 after nerve injury, and the expression returned to baseline by day 2. **g**, **h** qRT-PCR of sorted microglia from the lumbar spinal cord of adult *Cx3cr1-creER-yfp/+; +/+* animals (**g**) and qRT-PCR of RiboTag enriched microglia mRNA from lumbar spinal cord (**h**). *Myc* was transiently upregulated in the ipsilateral lumbar cord microglia on day 1 after nerve injury, and the expression returned to baseline by day 2 after nerve injury. Analyses are two-way ANOVA with Sidak’s multiple-comparison test, *n* = 4–6, means ± SEM, ****P < 0.0001; ns, not significant.

We then identified the cell-cycle state of each sequenced microglia cell by assessing its expression of specific cell-cycle marker genes^36,37^, followed by the visualization of these cells using linear-dimension reduction^38^. The scRNA-Seq principal component analysis (PCA) plots showed that most lumbar cord microglia in naive young adult *Cx3cr1-creER-yfp/+* heterozygous mice were in gap-1 (G1) phase of the cell cycle, with a very small proportion of cells in synthesis (S) or gap-2 (G2)/mitosis (M) phases (Fig. 1a), consistent with previous reports of a low-baseline microglia proliferation^5–7^. One day after nerve injury, a substantial portion of microglia was in S phase (Fig. 1b), whereas a substantial G2/M-phase population was observed by 2 and 3 days after nerve injury (Fig. 1c, d). Thus, our scRNA-Seq results recapitulated the previously reported observations that peripheral nerve injury triggered microglia proliferation in the spinal cord^9,21,27,30^.

### *Myc* is transiently upregulated in spinal lumbar cord microglia after sciatic nerve injury

When we examined the single-cell expression of *Mki67*, which encodes proliferation marker Ki67, with 10× Genomic Loupe Cell Browser, we found that more microglia cells expressed *Mki67* on day 2 after nerve injury (Fig. 1e), consistent with the previous reports that the proliferation of lumbar cord microglia begins 2 days after peripheral nerve injury^9,39^. We also found *Myc* transcripts, which encode the transcription factor MYC that is involved in regulating proliferation in many cell types^40^, in only a few microglia from naive animals, but in substantially more microglia 1 day after nerve injury (Fig. 1e), a time point preceding the induction of *Mki67* expression. We found that *Myc* induction was transient, the highest at day 1, and resolved by day 2 after nerve injury (Fig. 1f).

To validate our scRNA-Seq finding of *Myc* upregulation in microglia shortly after nerve injury, we FACS-sorted YFP (+) microglia from the ipsilateral (injury) and contralateral (uninjury) sides of lumbar spinal cord of young adult *Cx3cr1-creER-yfp/+* heterozygous mice 1 day and 2 days after nerve injury, and with quantitative RT-PCR (qRT-PCR) from these sorted cells, we confirmed that *Myc* was transiently upregulated in microglia of ipsilateral lumbar cord 1 day after nerve injury (Fig. 1g), when the expression of many microglia marker genes was unchanged (Supplementary Fig. S4). We also applied microglia RiboTag technique^41^ to investigate mRNA that specifically binds to microglia ribosomes, and we verified the transient upregulation of *Myc* in microglia of ipsilateral lumbar cord 1 day after nerve injury (Fig. 1h).

### Two phases of microglia proliferation

**Fig. 2:**
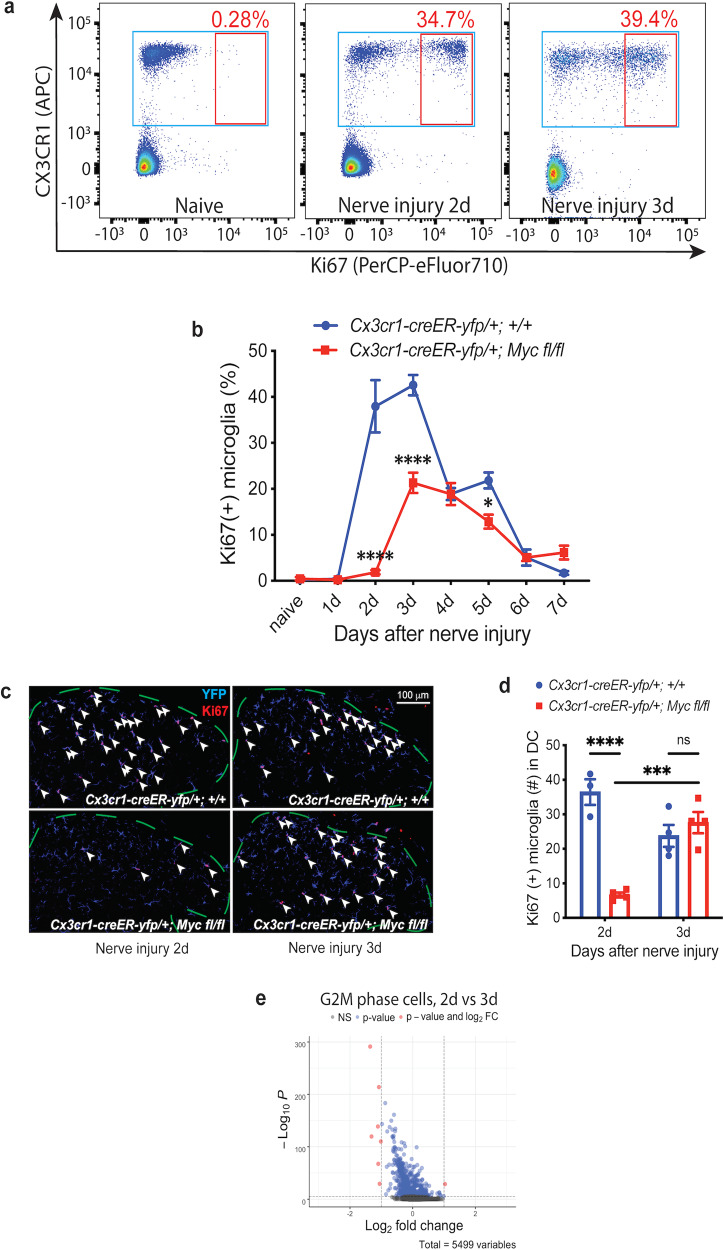
Microglia proliferation after nerve injury consists of a *Myc*-dependent early phase and a *Myc*-independent late phase. **a** Representative flow-cytometry scatter plots to assess microglia proliferation in lumbar spinal cord after sciatic nerve injury. The proportion of proliferating Ki67 (+) microglia in *Cx3cr1-creER-yfp/+; +/+* animals increased 2 and 3 days after nerve injury. **b** Quantification of flow cytometry highlights the time course of nerve injury-induced microglia proliferation. In control *Cx3cr1-creER-yfp/+; +/+* animals, microglia proliferation started at day 2, persisted through day 3, and then gradually reduced to termination by day 7 after nerve injury. In contrast, the early phase of microglia proliferation on day 2 after nerve injury was completely abolished in *Cx3cr1-creER-yfp/+; Myc fl/fl* cKO animals, but the late phase and the termination phase of microglia proliferation remained intact in cKO animals. **c**, **d** Representative Ki67 IHC staining (**c**) and the associated quantification (**d**) of lumbar dorsal cord microglia after nerve injury. Animals with *Myc* deletion from adult microglia had significantly less Ki67 (+) proliferating microglia in lumbar dorsal cord (DC) 2 days, but not 3 days, after nerve injury. The arrowheads point to the Ki67 (+) microglia, and the dashed lines outline the dorsal lumbar cord. Microglia are identified by YFP staining. **e** Differential expression volcano plot of pseudobulk scRNA-Seq analysis. The G2/M-phase microglia between day 2 and day 3 after nerve injury displayed distinct gene expression profiles. Analyses are two-way ANOVA with Sidak’s multiple-comparison test, *n* = 4–6, means ± SEM, *P < 0.05, ***P < 0.001, ****P < 0.0001, ns, not significant. The statistical analysis in **b** shows the difference between *Myc* cKO and control animals.

With flow cytometry, we characterized the time course of spinal cord microglia proliferation after sciatic nerve injury by analyzing the percentage of Ki67 (+) microglia, and observed that microglia proliferation started at day 2, maintained through day 3, and then gradually reduced to the termination of proliferation by day 7 after nerve injury (Fig. 2a, b), consistent with previous reports from the immunohistochemistry (IHC) studies^9,39^. To study the function of *Myc* in microglia proliferation, we deleted *Myc* from adult microglia by crossing *Myc*^*flox*^ mice^42^ with *Cx3cr1-creER-yfp* mice and then treated young adult *Cx3cr1-creER-yfp/+; Myc fl/fl* animals with tamoxifen. With flow cytometry, we found that *Myc* deletion from adult microglia completely prevented the early timepoint microglia proliferation 2 days after nerve injury (Fig. 2b; Supplementary Fig. S5), but microglia proliferation at later timepoints, as well as the termination of proliferation, remained (Fig. 2b). The same findings were echoed using IHC staining for Ki67 in the dorsal lumbar spinal cord: there was significantly less microglia proliferation in animals with *Myc* deletion specifically on day 2 after nerve injury, but the microglia proliferation 3 days after nerve injury was intact in *Myc* conditional-knockout (cKO) animals (Fig. 2c, d). Our IHC with EdU in lumbar dorsal cord further confirmed that *Myc* cKO mice had specific deficit in microglia proliferation 2 days, but not 3 days, after nerve injury (Supplementary Fig. S6).

We also counted the total number of microglia in lumbar dorsal cord of naive animals and of animals at different timepoints after nerve injury and found that although *Myc* deletion in adult microglia did not change the total number of microglia in naive animals, it resulted in significantly less dorsal cord microgliosis after nerve injury (Supplementary Fig. S7). Consistent with our observation that the early timepoint microlgia proliferation 2 days after nerve injury was abolished in *Myc* cKO mice (Fig. 2b–d), the total number of dorsal cord microglia remained unchanged in *Myc* cKO mice 2 days after nerve injury, whereas it started to increase in control mice at that timepoint (Supplementary Fig. S7b).

The aforementioned results suggest that nerve injury-induced microglia proliferation has two phases, a *Myc*-dependent early phase on day 2 after nerve injury, and a *Myc*-independent late phase on day 3 after nerve injury (Fig. 2b). Indeed, the PCA plot distribution of the G2/M-phase microglia 2 days after nerve injury was different from that of the G2/M microglia 3 days after nerve injury (Fig. 1c, d). A further comparison of the G2/M microglia between 2 and 3 days after nerve injury using a pseudobulk differential expression analysis^43^ highlighted the differences in gene expression profiles between these two populations (Fig. 2e; Supplementary Table S1).

### *Tnfaip3* is downregulated in spinal lumbar cord microglia after sciatic nerve injury

**Fig. 3:**
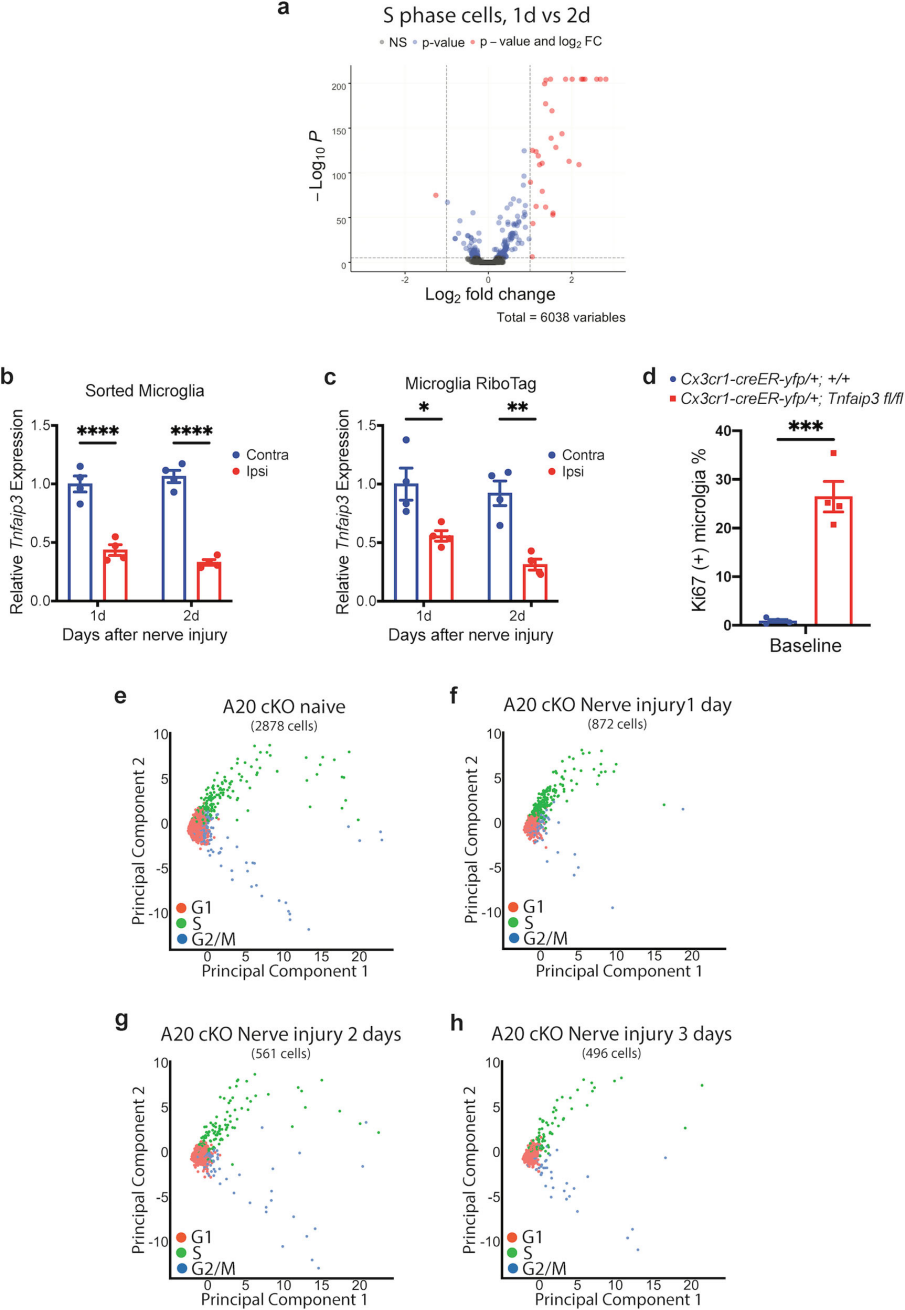
*Tnfaip3* is downregulated in microglia after nerve injury. **a** Differential expression volcano plot of pseudobulk scRNA-Seq analysis. The S-phase microglia from day 1 after nerve injury displayed different gene expression profile from that of the S-phase microglia 2 days after nerve injury in *Cx3cr1-creER-yfp/+; +/+* animals. **b**, **c** qRT-PCR of FACS-sorted microglia from lumbar spinal cord of *Cx3cr1-creER-yfp/+; +/+* animals (**b**) and of RiboTag enriched microglia mRNA from lumbar spinal cord (**c**). *Tnfaip3* was downregulated in the microglia of ipsilateral lumbar cord 1 and 2 days after nerve injury. **d** Quantification of proliferating Ki67 (+) microglia by flow cytometry. Naive uninjured *Cx3cr1-creER-yfp/+; Tnfaip3 fl/fl* A20 cKO animals had significantly more baseline microglia proliferation than those of naive control *Cx3cr1-creER-yfp/+; +/+* animals. **e** scRNA-Seq PCA scatter plot of naive *Cx3cr1-creER-yfp/+; Tnfaip3 fl/fl* A20 cKO microglia. The distributions of G1, S, and G2/M phase cells were similar to that of *Cx3cr1-creER-yfp/+; +/+* control animals 3 days after nerve injury as shown in Fig. 1d. **f**–**h** scRNA-Seq PCA scatter plot of *Cx3cr1-creER-yfp/+; Tnfaip3 fl/fl* A20 cKO microglia 1 day (**f**), 2 days (**g**), and 3 days (**h**) after nerve injury. The distributions of G1, S, and G2/M-phase cells were similar to that of naive *Cx3cr1-creER-yfp/+; Tnfaip3 fl/fl* A20 cKO animals (**e**). Analyses are two-way ANOVA with Sidak’s multiple comparison test (**b**, **c**) and unpaired two-tailed *t*-test (**d**), *n* = 4–6, means ± SEM, *P < 0.05, **P < 0.01, ***P < 0.001, and ****P < 0.0001.

The PCA plot distribution of the S-phase microglia 1 day after nerve injury was also notably different from that of the S-phase microglia 2 days after nerve injury (Fig. 1b, c), which reflects the different gene expression profiles of the S-phase microglia between the two timepoints (Fig. 3a; Supplementary Table S2). To further investigate the mechanism behind such difference, we turned to tumor-necrosis factor alpha-induced protein 3 (*Tnfaip3*), commonly known as A20, as *Tnfaip3* has been reported to inhibit microglia proliferation^44^. qRT-PCR of sorted microglia and microglia RiboTag revealed that *Tnfaip3* was downregulated in microglia of ipsilateral lumbar spinal cord, both 1 and 2 days after nerve injury (Fig. 3b, c). Of note, on day 2 after nerve injury when *Myc* expression had returned to baseline (Fig. 1g, h), *Tnfaip3* remained downregulated in microglia (Fig. 3b, c). With flow cytometry, we found that deleting *Tnfaip3* from adult microglia, by crossing *Tnfaip3*^*flox*^ mice^45^ with *Cx3cr1-creER-yfp* mice and then treating young adult *Cx3cr1-creER-yfp/+; Tnfaip3 fl/fl* animals with tamoxifen, significantly enhanced baseline microglia proliferation without any nerve injury (Fig. 3d; Supplementary Fig. S8a). Our IHC studies also showed that A20 cKO mice have increased microglia proliferation (Supplementary Fig. S9) and increased microglia number (Supplementary Fig. S10) in the lumbar spinal dorsal cord without nerve injury. These results indicate that A20 inhibits microglia proliferation, and nerve injury induced A20 downregulation to promote microglia proliferation.

To further investigate the function of *Tnfaip3* in microglia proliferation, we FACS-sorted YFP (+) cells from the parenchyma of lumbar spinal cords of young adult *Cx3cr1-creER-yfp/+; Tnfaip3 fl/fl* cKO mice at different timepoints after nerve injury for scRNA-Seq. We found that the microglia in naive *Tnfaip3* cKO mice had a substantial proportion of S-phase and G2/M-phase cells (Fig. 3e), consistent with our flow cytometry and IHC results of enhanced microglia proliferation in naive *Tnfaip3* cKO mice. Interestingly, the distribution of the S- and G2/M-phase cells in PCA plot of naive *Cx3cr1-creER-yfp/+; Tnfaip3 fl/fl* cKO mice without injury (Fig. 3e) was similar to that of *Cx3cr1-creER-yfp/+; +/+* control mice 3 days after nerve injury (Fig. 1d), but was quite different from that of *Cx3cr1-creER-yfp/+; +/+* animals 2 days after nerve injury (Fig. 1c).

### *Tnfaip3* mediates late phase of microglia proliferation

**Fig. 4:**
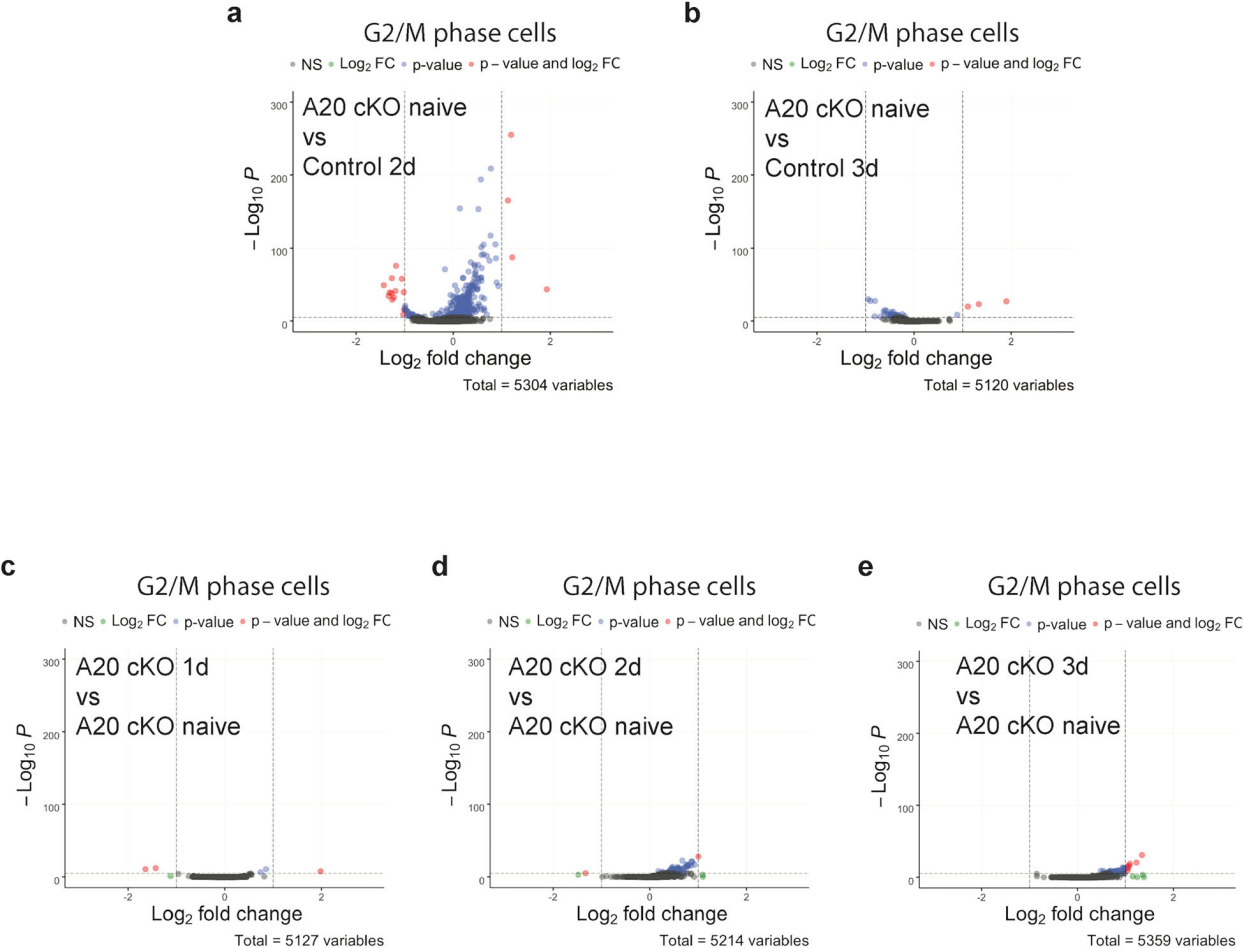
Differential expression volcano plots of pseudobulk scRNA-Seq showing that A20 naive cKO has similar G2/M microglia expression profile as control 3 days after nerve injury. **a** The G2/M-phase microglia of naive uninjured *Cx3cr1-creER-yfp/+; Tnfaip3 fl/fl* A20 cKO animals exhibited different gene expression profile from the G2/M phase microglia of control *Cx3cr1-creER-yfp/+; +/+* animals 2 days after nerve injury. **b** The G2/M-phase microglia of naive uninjured *Cx3cr1-creER-yfp/+; Tnfaip3 fl/fl*A20 cKO animals shared similar gene expression profile with the G2/M-phase microglia of control *Cx3cr1-creER-yfp/+; +/+* animals 3 days after nerve injury. **c**, **e** The gene expression profile of the G2/M-phase microglia from *Cx3cr1-creER-yfp/+; Tnfaip3 fl/fl* A20 cKO animals remained largely unchanged 1 day (**c**), 2 days (**d**), or 3 days (**e**) after nerve injury.

Because the G2/M microglia 2 days after nerve injury had different gene expression profile from the G2/M microglia 3 days after nerve injury (Fig. 2e), we compared the gene expression profile of the G2/M microglia from naive *Cx3cr1-creER-yfp/+; Tnfaip3 fl/fl* cKO mice without injury to the gene expression profiles of the G2/M microglia from control *Cx3cr1-creER-yfp/+; +/+* mice 2 days and 3 days after nerve injury. We found that the G2/M microglia of naive *Cx3cr1-creER-yfp/+; Tnfaip3 fl/fl* cKO mice had distinct gene expression profile from the G2/M microglia of *Cx3cr1-creER-yfp/+; +/+* mice 2 days after nerve injury (Fig. 4a; Supplementary Table S3). However, the naive *Tnfaip3* cKO G2/M microglia shared a much similar gene expression profile with the G2/M microglia of *Cx3cr1-creER-yfp/+; +/+* mice 3 days after nerve injury (Fig. 4b). These results suggested that the downregulation of *Tnfaip3* in adult microglia results in increased microglia proliferation with a transcriptomic signature resembling that of the late-phase microglia proliferation 3 days after nerve injury.

### The early phase G2/M microglia do not appear in *Tnfaip3* cKO mice after nerve injury

We found that the distributions of the S- and G2/M-phase cells in the PCA plot of *Cx3cr1-creER-yfp/+; Tnfaip3 fl/fl* cKO mice 1 day (Fig. 3f), 2 days (Fig. 3g), and 3 days (Fig. 3h) after nerve injury were all similar to that of naive *Cx3cr1-creER-yfp/+; Tnfaip3 fl/fl* cKO mice without injury (Fig. 3e) and that of *Cx3cr1-creER-yfp/+; +/+* mice 3 days after nerve injury (Fig. 1d). Similarly, a comparison of the G2/M microglia transcriptome profiles between *Cx3cr1-creER-yfp/+; Tnfaip3 fl/fl* cKO mice 1 day (Fig. 4c), 2 days (Fig. 4d), and 3 days (Fig. 4e) after nerve injury and the naive *Cx3cr1-creER-yfp/+; Tnfaip3 fl/fl* cKO mice showed that the G2/M microglia transcriptome profiles in *Tnfaip3* cKO remained relatively unchanged even after nerve injury. These results suggest that the early phase G2/M microglia 2 days after nerve injury in control animals did not appear in *Tnfaip3* cKO mice after nerve injury.

### Cyclin-dependent kinase 1 (CDK1) in early phase microglia proliferation

**Fig. 5:**
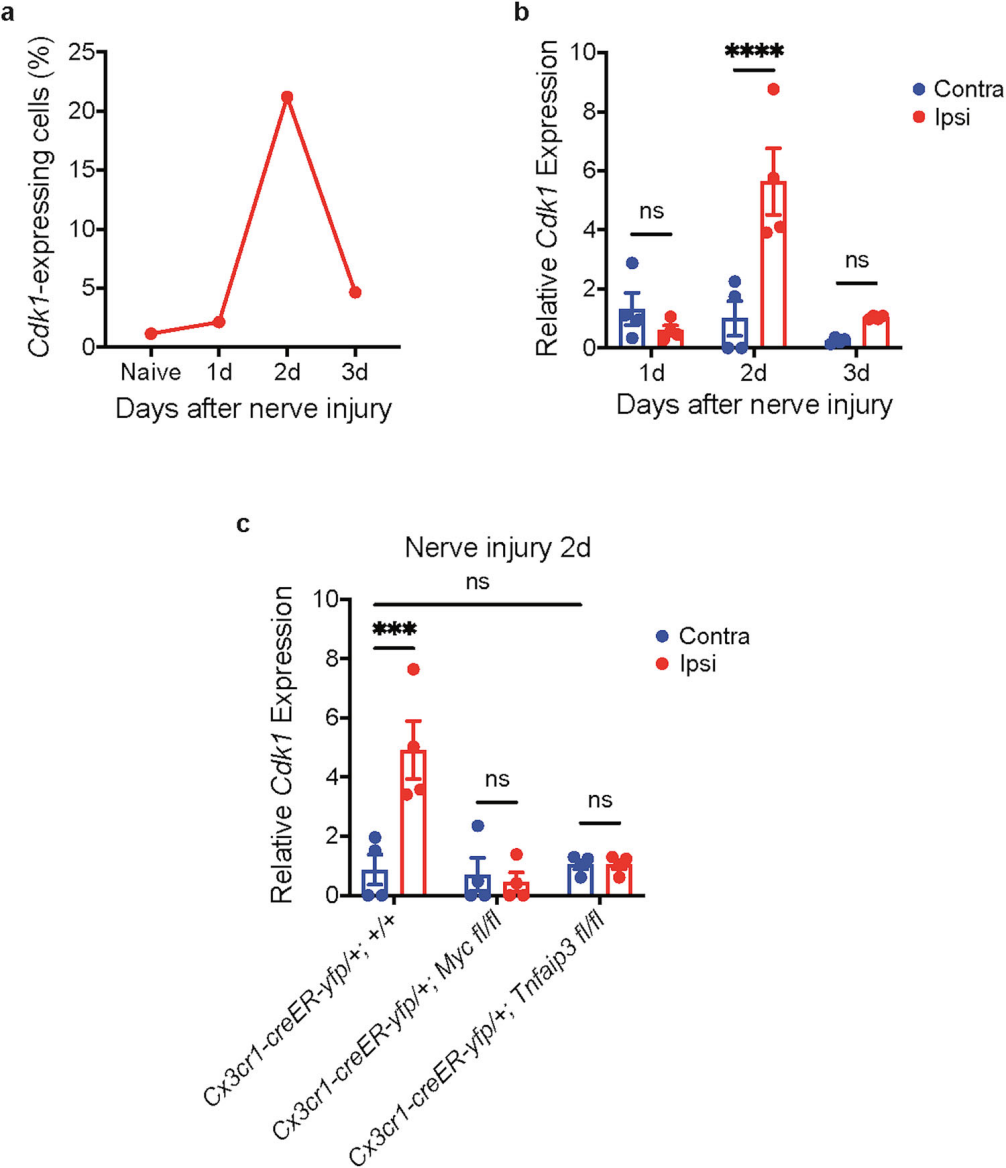
Signaling pathways in microglia proliferation. **a** Proportion of microglia cells expressing *Cdk1* from scRNA-Seq analysis. *Cdk1* was expressed in a higher proportion of microglia 2 days after nerve injury. **b** qRT-PCR of sorted microglia from lumbar spinal cord of *Cx3cr1-creER-yfp/+; +/+* animals. *Cdk1* was upregulated in microglia from ipsilateral lumbar cord 2 days, but not 1 day or 3 days, after nerve injury. **c** qRT-PCR of sorted lumbar cord microglia. *Cdk1* upregulation in microglia 2 days after nerve injury was prevented when *Myc* was deleted from adult microglia. Deleting *Tnfaip3* from adult microglia did not induce *Cdk1* upregulation. Deletion of *Tnfaip3* also prevented *Cdk1* upregulation 2 days after nerve injury. Analyses are two-way ANOVA with Sidak’s multiple-comparison test, *n* = 4–6, means ± SEM, ***P < 0.001, ****P < 0.0001; ns, not significant.

Our results have demonstrated that the nerve injury-induced microglia proliferation had a *Myc* upregulation-mediated early phase and a *Tnfaip3* downregulation-mediated late phase. To further explore the mechanism of microglia proliferation, we turned to CDK1, a catalytic subunit of the M phase-promoting factor that has crucial function in M phase of the cell cycle^46^. Our analysis of scRNA-Seq data of *Cx3cr1-creER-yfp/+; +/+* mice with 10 × Genomic Loupe Cell Browser revealed that more microglia expressed *Cdk1* on day 2 after nerve injury (Fig. 5a), a timepoint of early phase *Myc*-dependent microglia proliferation. However, the expression of *Cdk1* reduced remarkably by 3 days after nerve injury (Fig. 5a), a timepoint of late-phase *Myc*-independent microglia proliferation (Fig. 2b).

Our qRT-PCR of sorted microglia from *Cx3cr1-creER-yfp/+; +/+* mice confirmed that *Cdk1* was upregulated in the ipsilateral lumbar spinal cord 2 days after nerve injury, and its expression returned to baseline on day 3 after nerve injury (Fig. 5b). This result suggests that *Cdk1* was induced only in the early phase microglia proliferation. In line with the previous report that *Myc* stimulates cell-cycle progression by activating CDK1^47^, we found that deleting *Myc* from adult microglia (*Cx3cr1-creER-yfp/+; Myc fl/fl*) completely prevented the *Cdk1* upregulation 2 days after nerve injury (Fig. 5c), indicating that upregulation of *Cdk1* in microglia is downstream of *Myc*.

Along with our observation that *Cdk1* was not upregulated in microglia 3 days after nerve injury (Fig. 5a, b), we found that deleting *Tnfaip3* from adult microglia (*Cx3cr1-creER-yfp/+; Tnfaip3 fl/fl*) did not cause *Cdk1* upregulation (Fig. 5c). These results suggest that *Cdk1* is specifically involved in the *Myc*-dependent early phase, but not in the *Tnfaip3*-mediated late phase, of microglia proliferation. We also found that the *Cdk1* upregulation 2 days after nerve injury did not occur in *Tnfaip3* cKO (Fig. 5c), consistent with our scRNA-seq analyses that the nerve injury-induced early phase microglia proliferation did not occur in *Tnfaip3* cKO mice (Figs. 3e–h, 4).

### Interactions between *Myc* and *Tnfaip3* pathways

**Fig. 6:**
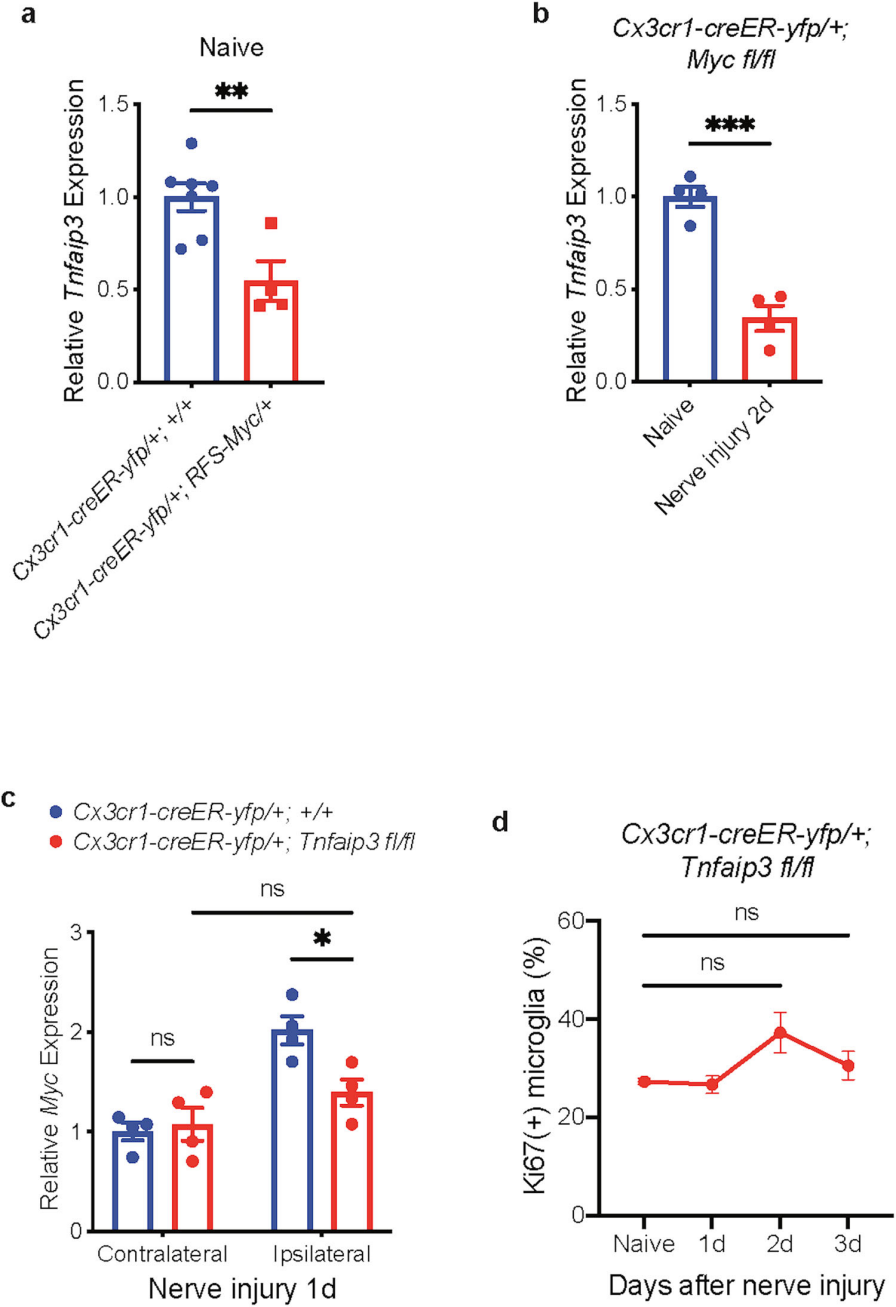
Interaction between *Myc* upregulation and *Tnfaip3* downregulation in microglia. **a** qRT-PCR of sorted lumbar cord microglia. Overexpression of *Myc* in microglia of adult naive *Cx3cr1-creER-yfp/+; RFS-Myc/+* animals resulted in downregulation of *Tnfaip3*. **b** qRT-PCR of sorted lumbar cord microglia from *Cx3cr1-creER-yfp/+; Myc fl/fl* cKO animals. Deletion of *Myc* from adult microglia did not prevent *Tnfaip3* downregulation 2 days after nerve injury. **c** qRT-PCR of sorted lumbar cord microglia. Deletion of *Tnfaip3* from adult microglia prevented *Myc* upregulation 1 day after nerve injury. **d** Quantification of Ki67 flow cytometry. The proliferation of lumbar cord microglia in *Cx3cr1-creER-yfp/+; Tnfaip3 fl/fl* cKO animals did not increase after nerve injury. Analyses are unpaired two-tailed *t*-test (**a**, **b**) and one-way ANOVA with Tukey’s multiple-comparison test (**c**, **d**), *n* = 4–6, means ± SEM, *P < 0.05, **P < 0.01, ***P < 0.001; ns, not significant.

To study the potential interaction between *Myc* and *Tnfaip3* pathways, we crossed *Cx3cr1-creER-yfp* mice with *ROSA-Floxed-Stop (RFS)-Myc* mice^48^, in which the treatment of young adult *Cx3cr1-creER-yfp/+; RFS-Myc/+* mice with tamoxifen resulted in the upregulation of *Myc* in adult microglia (Supplementary Fig. S11). We found that *Myc* upregulation produced *Tnfaip3* downregulation in microglia of naive animals without any injury (Fig. 6a), suggesting that *Myc* upregulation in microglia may contribute to the *Tnfaip3* downregulation after nerve injury. However, microglia *Tnfaip3* downregulation 2 days after nerve injury was intact in *Cx3cr1-creER-yfp/+; Myc fl/fl* cKO animals (Fig. 6b), indicating that *Myc* was not required for nerve injury-induced *Tnfaip3* downregulation in microglia. On the other hand, although deleting *Tnfaip3* from adult microglia in *Cx3cr1-creER-yfp/+; Tnfaip3 fl/fl* cKO did not change the baseline *Myc* expression in spinal cord microglia, it abolished the nerve injury-induced *Myc* upregulation in microglia of spinal cord one day after nerve injury (Fig. 6c), hinting that *Tnfaip3* downregulation suppresses *Myc* upregulation in microglia after nerve injury. This result also explains our previous results that the *Myc*-dependent early phase microglia proliferation and *Cdk1* upregulation 2 days after nerve injury did not occur in *Cx3cr1-creER-yfp/+; Tnfaip3 fl/fl* cKO mice (Figs. 3e–h, 4, 5c). Indeed, our flow cytometry (Fig. 6d; Supplementary Fig. S8b) study showed no changes in microglia proliferation beyond baseline levels in *Tnfaip3* cKO mice after nerve injury. Our IHC studies confirmed that A20 cKO mice did not have increased microglia proliferation (Supplementary Fig. S12) or any increase in microglia number (Supplementary Fig. S13) in lumbar spinal dorsal cord after nerve injury.

### Termination of induced microglia proliferation

**Fig. 7:**
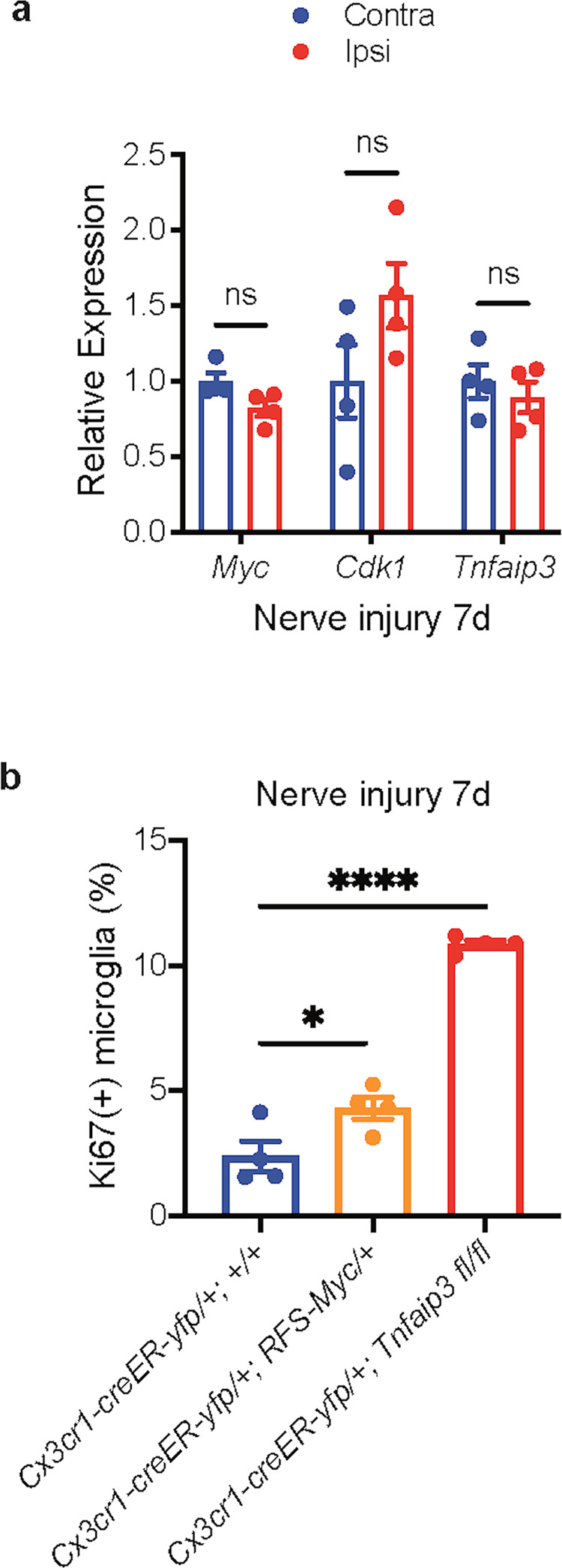
The termination of microglia proliferation. **a** qRT-PCR of sorted lumbar cord microglia from *Cx3cr1-creER-yfp/+; +/+* animals. The expression levels of *Myc, Cdk1*, and *Tnfaip3* of lumbar cord microglia returned to control contralateral levels 7 days after nerve injury. **b** Quantification of Ki67 flow cytometry. Overexpression of *Myc* in adult microglia or deletion of *Tnfaip3* from adult microglia prevented the termination of lumbar cord microglia proliferation 7 days after nerve injury. Analyses are one-way ANOVA with Tukey’s multiple-comparison test (**b**) or two-way ANOVA with Sidak’s multiple-comparison test (**a**), *n* = 4–6, means ± SEM, *P < 0.05, **P < 0.01, ***P < 0.001; ns, not significant.

Although it is important for microglia to expand their population by proliferation in response to various pathological stimulations, it is equally crucial for microglia to terminate their induced proliferation to prevent cancer-like uncontrollable cell growth. However, the mechanism for the termination of microglia proliferation is almost completely unknown. Even though the proliferative signal released by injured sensory neurons persists for at least several weeks^9,49^, the nerve injury-induced microglia proliferation only lasts for less than 7 days (Fig. 2b). When microglia proliferation has been terminated by day 7 after nerve injury, with qRT-PCR of sorted lumbar cord microglia, we found that the expression of *Myc, Cdk1*, and *Tnfaip3* had returned to baseline (Fig. 7a). Moreover, our flow-cytometry study revealed that the persistent *Myc* upregulation (*Cx3cr1-creER-yfp/+; RFS-Myc/+*) or *Tnfaip3* downregulation (*Cx3cr1-creER-yfp/+; Tnfaip3 fl/fl*) in adult microglia prevented the termination of microglia proliferation 7 days after nerve injury (Fig. 7b). Thus, the termination of nerve injury-induced microglia proliferation requires both *Myc* and *Tnfaip3* to resume their baseline expression.

### The *Myc* upregulation-mediated early phase is not sufficient to induce *Tnfaip3* downregulation-mediated late-phase proliferation

**Fig. 8:**
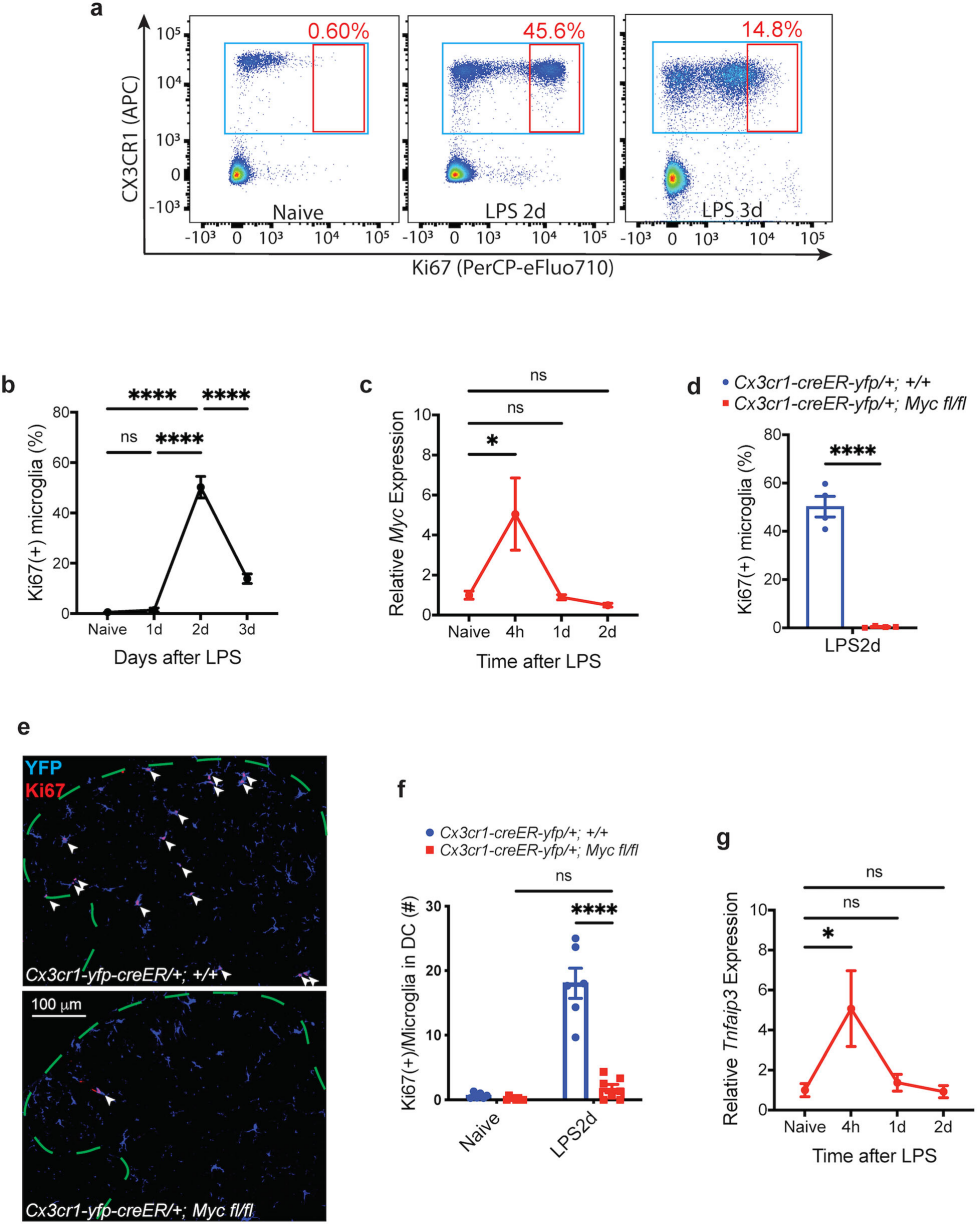
*Myc*-dependent early phase microglia proliferation is not sufficient to induce late-phase microglia proliferation. **a**, **b** Ki67 flow-cytometry scatter plots (**a**) and the associated quantification (**b**). Ki67 (+) proliferating microglia increased transiently in the spinal cord of *Cx3cr1-creER-yfp/+; +/+* animals 2 days after LPS treatment. **c** qRT-PCR of sorted lumbar cord microglia from *Cx3cr1-creER-yfp/+; +/+* animals. *Myc* was transiently upregulated in microglia 4 h after LPS treatment, and the expression returned to baseline level by 1 day. **d** Quantification of Ki67 flow cytometry. *Myc* deletion in adult microglia abolished the LPS-induced lumbar cord microglia proliferation. **e**, **f** Representative Ki67 IHC images (**e**) and the associated quantification (**f**). Animals with *Myc* deletion from adult microglia had significantly less Ki67 (+) proliferating microglia in lumbar dorsal cord (DC) 2 days after LPS. The arrowheads point to the Ki67 (+) microglia, and the dashed lines outline the dorsal lumbar cord. Microglia are identified by YFP staining. **g** qRT-PCR of sorted lumbar cord microglia from *Cx3cr1-creER-yfp/+; +/+* animals. *Tnfaip3* was transiently upregulated in lumbar cord microglia 4 h after LPS treatment, and the expression returned to baseline level by 1 day, without any *Tnfaip3* downregulation. Analyses are one-way ANOVA with Tukey’s multiple-comparison test (**b**, **c**, **g**) or two-way ANOVA with Sidak’s multiple-comparison test (**f**), and unpaired two-tailed *t*-test (**d**), *n* = 4–6, means ± SEM, *P < 0.05, **P < 0.01, ***P < 0.001; ns, not significant.

To further investigate the mechanism of microglia proliferation, we turned to lipopolysaccharide (LPS), an endotoxin from Gram-negative bacteria that can produce microglia proliferation in mouse brain by a single peritoneal injection of 0.5–2.5 mg/kg^22^. With flow cytometry, we found that a single peritoneal injection of 2.5 mg/kg LPS induced microglia proliferation in mouse spinal cord (Fig. 8a, b) and multiple brain regions (Supplementary Fig. S14a) 2 days after the treatment. With CCR2-RFP mice, in which all the peripheral monocytes are labeled with RFP^35^, we found no RFP+ monocyte infiltration into the spinal cord after LPS injection (data not shown), suggesting that the expansion of microglia population after LPS treatment was the result of microglia proliferation. In agreement with the nerve-injury model, our qRT-PCR of sorted microglia showed that *Myc* was also transiently upregulated in the microglia of spinal cord (Fig. 8c) and various brain regions (Supplementary Fig. S14b) after LPS treatment. Our flow cytometry (Fig. 8d) and IHC studies (Fig. 8e, f; Supplementary Fig. S15) on animals with *Myc* deletion from adult microglia (*Cx3cr1-creER-yfp/+; Myc fl/fl*) revealed that *Myc* is necessary for LPS-induced microglia proliferation in spinal cord, as well as in many brain regions (Supplementary Fig. S14c).

However, the LPS-induced microglia proliferation was short-lived and lasted for only one day (Fig. 8a, b), unlike the prolonged microglia proliferation seen after nerve injury (Fig. 2b). In addition, contrary to the nerve-injury model, we found that LPS treatment did not induce any *Tnfaip3* downregulation (Fig. 8g), which we showed to mediate the late-phase of proliferation. Thus, the transient *Myc*-dependent microglia proliferation induced by LPS was not followed by *Tnfaip3-*mediated late-phase microglia proliferation. In other words, the *Myc* upregulation-mediated early phase of microglia proliferation by itself is not sufficient to induce the late phase proliferation. Given that the *Myc* upregulation-mediated early phase of proliferation is not required for the late phase proliferation either (Fig. 2b), our results demonstrate that the early phase of microglia proliferation is neither necessary nor sufficient for the late phases of microglia proliferation.

## Discussion

### Microglia proliferation has an early phase, a late phase, and a termination phase

In a nerve-injury-induced microglia proliferation model, we confirmed the previous reports^9,39^ that microglia proliferation starts on day 2, maintains on day 3, and gradually reduces to termination by day 7 after nerve injury (Fig. 2b). We also found that the early phase of microglia proliferation 2 days after nerve injury did not occur when *Myc* was deleted from adult microglia, with relatively intact late phase of microglia proliferation 3 days after nerve injury and the termination phase of microglia proliferation 6–7 days after nerve injury (Fig. 2b). Thus, the nerve-injury-induced microglia proliferation has a *Myc*-dependent early phase and a *Myc*-independent late phase.

### The proliferating microglia at different timepoints after nerve injury have different gene expression profiles

Our observation that microglia proliferation has distinct early and late phases is further supported by our scRNA-seq study. By analyzing the expression of cell-cycle marker genes^36,37^, we assigned each sequenced microglia cell into specific cell cycles. We found that the G2M microglia cells in the early phase (2 days after nerve injury) had different gene expression profile from the G2M cells in the late phase (3 days after nerve injury) (Fig. 2e). Similarly, we found that the S-microglia cells 1 day after nerve injury were also different from the S cells 2 days after nerve injury (Fig. 3a).

### Myc–CDK1 pathway mediates the early phase of microglia proliferation

CDK1 is a serine/threonine kinase that plays a crucial role in driving the M phase in mitosis, and its activity is increased only in the M phase of the cell cycle^46^. Both of our scRNA-Seq and qRT-PCR results showed that *Cdk1* was upregulated specifically in microglia of *Myc*-dependent early phase (day 2 after nerve injury), but not in microglia of *Myc*-independent late phase (day 2 after nerve injury) (Fig. 5a, b), further supporting our observations that the early phase and the late phase of microglia proliferation are distinct with different mechanisms. Moreover, we found that the *Cdk1* upregulation itself was *Myc*-dependent in microglia (Fig. 5c). Thus, our results demonstrate that the *Myc*–CDK1 pathway mediates the early phase of microglia proliferation. Further studies are needed to investigate how *Myc* regulates *Cdk1* expression in microglia and how CDK1 regulates the early phase of microglia proliferation.

### A20 mediates the late phase of microglia proliferation

A20, encoded by *Tnfaip3* gene, is a key player in ubiquitin-dependent signals, and it participates in regulating diverse signaling pathways^50^. Our results that deletion of *Tnfaip3* from adult microglia resulted in microglia proliferation in naive animals (Fig. 3d) and that *Tnfaip3* was downregulated in microglia after nerve injury (Fig. 3b, c) suggested that A20 inhibits microglia proliferation, and that nerve injury suppressed the inhibitory effect of A20 to promote microglia proliferation. Importantly, our scRNA-seq analysis showed that the proliferating G2/M microglia in naive *Tnfaip3* cKO without injury was similar to the G2/M microglia of the late-phase proliferation 3 days after nerve injury, but different from the G2/M microglia of the early phase 2 days after nerve injury (Fig. 4), suggesting that suppression of A20 mediates the late-phase microglia proliferation. A20 has been implicated in both promoting and inhibiting proliferation in a cell-type-specific context^51,52^, but how A20 regulates the late phase of microglia proliferation remains to be further studied.

### The early phase is neither necessary nor sufficient for the late phases of microglia proliferation

Although the early phase precedes the late-phase microglia proliferation, our results demonstrated that the late-phase proliferation occurred independently from the early phase, further supporting that the mechanisms for the early phase and the late phase of microglia proliferation are different. In *Myc* cKO animals, *Tnfaip3* was downregulated in microglia to the level similar as in control animals after nerve injury (Figs. 6b, 3b), and the late-phase microglia proliferation occurred without the precedent early phase proliferation (Fig. 2b), suggesting that the early phase was not required for the development of the late-phase microglia-proliferation. On the other hand, in LPS-induced microglia proliferation model, the *Myc*-dependent transient microglia proliferation 2 days after LPS treatment was not followed by *Tnfaip3*-downregulation-mediated late-phase proliferation (Fig. 8b), suggesting that the early phase was not sufficient for the development of the late-phase microglia proliferation either.

### The late phase suppresses the early phases of microglia proliferation

In contrast, our results showed that the *Tnfaip3*-downregulation-mediated late-phase proliferation inhibited the *Myc*-upregulation-mediated early phase proliferation. We found that genetic deletion of *Tnfaip3* from adult microglia completely blocked the nerve-injury-induced upregulation of *Myc* (Fig. 6c) or *Cdk1* (Fig. 5c) in microglia. Moreover, our scRNA-seq results revealed that the early phase-specific G2/M microglia cells never appeared in *Tnfaip3* cKO after nerve injury (Fig. 4). Thus, the late-phase microglia proliferation not only occurred independently from the early phase, but also suppressed the early phase proliferation.

### Termination of microglia proliferation

We and others have previously demonstrated that peripheral nerve injury induces the injured dorsal root ganglion (DRG) sensory neurons to express colony-stimulating factor 1 (CSF1)^9,49^, a cytokine critical for microglia development^1^, and CSF1 is subsequently transported from the injured DRG neurons to the spinal cord to stimulate microglia proliferation^9^. Interestingly, although the nerve-injury-induced CSF1 expression in the injured DRG neurons lasts for at least several weeks^9,49^, the nerve-injury-induced spinal cord microglia proliferation only lasts for less than one week^39^ (Fig. 2b). We now show that when microglia proliferation was terminated at 7 days after nerve injury, the expression of both *Myc* and *Tnfaip3* in microglia had returned to baseline (Fig. 7a), and this resumption of baseline expression of *Myc* and *Tnfaip3* was necessary for the termination of nerve-injury-induced microglia proliferation (Fig. 7b). How *Myc* and *Tnfaip3* resume their baseline expression needs to be further studied.

### Mechanisms of microglia proliferation

In summary, our results suggest that mature differentiated microglia proliferate with two distinct phases mediated by two different mechanisms (Supplementary Fig. S16). Nerve injury leads to transient *Myc* upregulation in microglia, which in turn induces *Cdk1* upregulation to mediate an early phase of microglia proliferation. In parallel, nerve injury also induces sustained *Tnfaip3* downregulation in microglia, which mediates the late-phase microglia proliferation. Although the early phase proliferation is neither necessary nor sufficient for the late-phase proliferation, *Myc* upregulation enhances *Tnfaip3* downregulation, whereas *Tnfaip3* downregulation suppresses *Myc* upregulation and the subsequent *Cdk1* upregulation. However, because the *Myc* upregulation after nerve injury is transient, such transient *Myc* upregulation is unlikely to produce sustained *Tnfaip3* downregulation for late-phase microglia proliferation. The termination of microglia proliferation requires both *Myc* and *Tnfaip3* to resume their baseline expression levels. Thus, we have delineated the signaling network in the distinct phases of microglia proliferation. To our knowledge, this is the first report on the separate phases of proliferation in fully differentiated cells.

## Materials and methods

### Animal lines

Animal experiments were approved by UCSF Institutional Animal Care and Use Committee and were conducted in accordance with the NIH Guide for the Care and Use of Laboratory animals. C57BL/6 wild-type, *Cx3cr1-creER-yfp*^32^ and RiboTag^53^ mice were purchased from Jackson Laboratory. *Myc*^*flox*^*, Tnfaip3*^*flox*^, and *ROSA-Floxed-Stop (RFS)-Myc* mice were described previously^42,45,48,54^. The experiments were conducted with roughly equal number of male and female young/adult animals.

### Surgeries and treatments

Sciatic-nerve transection and ligation was performed as previously described^9^. LPS (*E. coli O127:B8*, Sigma) was administered as a single 2.5 mg/kg intraperitoneal dose. Tamoxifen (Sigma) was injected as 100 mg/kg per animal for five consecutive days for recombination. All the experiments were performed 7 days after the first dose of tamoxifen injection.

### Microglia isolation

Mice were anesthetized with Avertin (Sigma) 25 mg per animal by intraperitoneal injection. After perfusion of 1× HBSS (Hank’s Balanced Salt Solution, Gibco), brain or spinal cord were collected freshly, with meningeal membrane removed. All the fresh samples were homogenized with 1× HBSS in Dounce homogenizer (DWK Life Science). The cell suspensions were then filtered through 70 mm cell strainer and washed with 1× HBSS, followed by mixture with Percoll (Sigma) gradient in HBSS with the final Percoll concentration of 33%. Samples were then centrifuged at 800 relative centrifugal force (RCF) for 20 min with no brake. Myelin and supernatant were removed by vacuum aspiration. Pellets, which contain isolated microglia, were collected and resuspended in 1 × HBSS.

### Fluorescence-activated cell sorting (FACS)

Freshly isolated microglia were sorted by FACS machine (BD FACSAria2) using 85um nozzle. After size selection and doublet exclusion, YFP (+) cells were sorted (1) directly into lysis buffer (QIAGEN), and RNA was isolated by RNeasy Plus Micro Kit (QIAGEN) following the manufacturer’s protocol for RNA collection or (2) into optimized serum-free media for single-cell RNA sequencing.

### Flow cytometry

Isolated microglia samples were first treated with Fc blocker (BD Pharmingen), then stained with surface CX3CR1-APC (1:500, Biolegend) and LIVE/DEAD Fixable Near-IR Dead Cell Staining Kit (1:2000, Invitrogen). The cells were then washed, fixed, and permeabilized with FoxP3/Transcription factor staining set (ThermoFisher), and then stained with Ki67–PerCP–eFluor710 (1:2000, Invitrogen). Flow cytometry was performed with a BD FACS Canto II and the results were analyzed by Flowjo software v10.6.1. Our gating strategy is similar as previously reported^55^ and presented in Supplementary Fig. S3.

### Immunohistochemistry

Immunohistochemistry was performed as previously described^9^, with the following antibodies: GFP (Abcam), Ki67 (Abcam), and CD11b (Abcam). Confocal images were collected with a Carl Zeiss LSM 700 microscope and were processed with Fiji/ImageJ (NIH).

### RiboTag immunoprecipitation

RiboTag immunoprecipitation was performed as previously described^41^. In short, the tissue of interest was dissected into homogenization buffer with 3% weight per volume ratio. Homogenized samples were centrifuged at 10,000 RCF for 10 min, and the supernatant was carefully separated for immunoprecipitation. The sample was incubated with anti-HA antibody (1:500, Biolegend) for 4 h. Protein A/G magnetic beads (Thermofisher Scientific) were prewashed with homogenization buffer, then added to the sample, and incubated overnight. The beads were collected by magnetic separation and then washed in high-salt buffer before being resuspended in lysis buffer for RNA isolation using RNeasy Plus Micro Kit (QIAGEN).

### Single-cell sequencing

Microglia sample preparation was performed as in our FACS experiments. Lumbar spinal cords of three animals were pooled per group, and the cells were harvested from naive animals and from animals 24 h, 48 h, or 72 h after nerve injury. Cell pellets were resuspended in optimized serum-free media previously described for improved microglia viability^56^: human recombinant TGF-β_2_ (2 ng/mL, Peprotech), murine IL34 (100 ng/mL, R&D Systems), and cholesterol (1.5 µg/mL, Avanti Polar Lipids). Single-cell libraries were generated using Chromium Single Cell 3′ Reagent Kit v3 (10× Genomics) by the UCSF Genomics Core^57^. The libraries were sequenced using Illumina NovaSeq 6000 according to the following protocol: read 1 (26 cycles, 16-bp cell barcode, 10-bp UMI), i7 (8 cycles, Illumina i7 sample index), and read 2 (91 cycles, transcript insert). The raw data have been uploaded to GEO (GSE 163948). The secure token for the reviewers is “irodugcozfudrmx”.

### scRNA-Seq data analysis

scRNA-Seq analysis was performed using RStudio (v1.3)^58^ with the Seurat package (V3.2.1)^38,59^. In brief, cells were filtered based on genes per cell, UMIs per cell, and percentage of mitochondrial gene reads per cell. The data were log-normalized and scaled. The scRNA-Seq from each condition was integrated to remove batch effects. Each cell was assigned a score, based on its expression of G2/M- and S-phase markers, linear-dimension reduction was performed subsequently via principal component analysis on cell-cycle genes to visualize the scRNA-Seq data^36,37^. To perform the pseudobulk differential expression comparison analysis^43^, two clusters of cells of interest were first selected and differentially expressed genes were compared based on the negative binomial distribution model and visualized as volcano plots. The scRNA-Seq data were also analyzed by 10× Genomic Loupe Cell Browser.

### Quantitative reverse-transcription polymerase-chain reaction

Total RNA was extracted with RNeasy Plus Micro Kit (QIAGEN) and cDNA was synthesized with SuperScript kit (Invitrogen). qRT-PCR was performed as previously described^9^ with Power iTag SYBR Green SuperMix (Bio-Rad) and specific primers in CFX384 Real-Time System (Bio-Rad). All primers were designed from the NCBI Primer-BLAST program. β-actin was used as the internal control for all the samples. The primer pairs used are

M b-actin F: CCACACCCGCCACCAGTTCG

M b-actin R: TACAGCCCGGGGAGCATCGT

cMyc 3′-F: AAAACAACGAAAAGGCCCCC

cMyc 3′-R: TTCAGAGGTGAGCTTGTGCT

qRT-PCR was also performed using the following TaqMan Gene Expression Assay probes with the TaqMan Universal PCR Master Mix and performed in CFX384 Real-Time System (Bio-Rad) according to the manufacturer’s suggested protocol (Thermo Fisher):

Actin: Mm02619580_g1

Myc: Mm00487804_m1

Tnfaip3: Mm00437121_m1

Cdk1: Mm00772472_m1

Hexb: Mm01282432_m1

Cx3cr1: Mm02620111_s1

Csf1r: Mm01266652_m1

P2ry12: Mm01950543_s1

### Statistical analysis

The measurements were taken from distinct samples. Two-tailed Student’s *t*-test was used to compare means of two groups, and one-way or two-way ANOVA tests were used for multiple comparisons with GraphPad Prism 9.0.1 (GraphPad Software, San Diego). Data are presented as means ± SEM. The data distribution was assumed to be normal and variances were assumed to be equal across the groups, but this was not formally tested.

## Supplementary information

Supplementary Information

## Data Availability

Microglia single-cell sequencing data are available at GEO (GSE 163948), with reviewers’ token of “irodugcozfudrmx”.

## References

[1] Ginhoux F (2010). Fate mapping analysis reveals that adult microglia derive from primitive macrophages. Science.

[2] Schulz C (2012). A lineage of myeloid cells independent of Myb and hematopoietic stem cells. Science.

[3] Salter MW, Beggs S (2014). Sublime microglia: expanding roles for the guardians of the CNS. Cell.

[4] Rossi F, Lewis C (2018). Microglia’s heretical self-renewal. Nat. Neurosci..

[5] Fuger P (2017). Microglia turnover with aging and in an Alzheimer’s model via long-term in vivo single-cell imaging. Nat. Neurosci..

[6] Reu P (2017). The lifespan and turnover of microglia in the human brain. Cell Rep..

[7] Tay TL (2017). A new fate mapping system reveals context-dependent random or clonal expansion of microglia. Nat. Neurosci..

[8] Askew K (2017). Coupled proliferation and apoptosis maintain the rapid turnover of microglia in the adult brain. Cell Rep..

[9] Guan Z (2016). Injured sensory neuron-derived CSF1 induces microglial proliferation and DAP12-dependent pain. Nat. Neurosci..

[10] Olmos-Alonso A (2016). Pharmacological targeting of CSF1R inhibits microglial proliferation and prevents the progression of Alzheimer’s-like pathology. Brain.

[11] Kraft AD, Kaltenbach LS, Lo DC, Harry GJ (2012). Activated microglia proliferate at neurites of mutant huntingtin-expressing neurons. Neurobiol. Aging.

[12] Spiller KJ (2018). Microglia-mediated recovery from ALS-relevant motor neuron degeneration in a mouse model of TDP-43 proteinopathy. Nat. Neurosci..

[13] Li T (2013). Proliferation of parenchymal microglia is the main source of microgliosis after ischaemic stroke. Brain.

[14] Bellver-Landete V (2019). Microglia are an essential component of the neuroprotective scar that forms after spinal cord injury. Nat. Commun..

[15] Ritzel RM (2019). Old age increases microglial senescence, exacerbates secondary neuroinflammation, and worsens neurological outcomes after acute traumatic brain injury in mice. Neurobiol. Aging.

[16] Ajami B, Bennett JL, Krieger C, McNagny KM, Rossi FM (2011). Infiltrating monocytes trigger EAE progression, but do not contribute to the resident microglia pool. Nat. Neurosci..

[17] Jordao, M. J. C. *et al*. Single-cell profiling identifies myeloid cell subsets with distinct fates during neuroinflammation. *Science*, **363**, (6425):eaat7554. 10.1126/science.aat7554 (2019). 10.1126/science.aat7554 30679343

[18] Feng L (2019). Microglial proliferation and monocyte infiltration contribute to microgliosis following status epilepticus. Glia.

[19] Hu X (2008). Macrophage antigen complex-1 mediates reactive microgliosis and progressive dopaminergic neurodegeneration in the MPTP model of Parkinson’s disease. J. Immunol..

[20] Yuan TF, Liang YX, Peng B, Lin B, So KF (2015). Local proliferation is the main source of rod microglia after optic nerve transection. Sci. Rep..

[21] Gu N (2016). Spinal microgliosis due to resident microglial proliferation is required for pain hypersensitivity after peripheral nerve injury. Cell Rep..

[22] Shankaran M (2007). Measurement of brain microglial proliferation rates in vivo in response to neuroinflammatory stimuli: application to drug discovery. J. Neurosci. Res..

[23] Gomez-Nicola D, Fransen NL, Suzzi S, Perry VH (2013). Regulation of microglial proliferation during chronic neurodegeneration. J. Neurosci..

[24] Nixon K, Kim DH, Potts EN, He J, Crews FT (2008). Distinct cell proliferation events during abstinence after alcohol dependence: microglia proliferation precedes neurogenesis. Neurobiol. Dis..

[25] Han W (2016). Cranial irradiation induces transient microglia accumulation, followed by long-lasting inflammation and loss of microglia. Oncotarget.

[26] Lehmann ML, Cooper HA, Maric D, Herkenham M (2016). Social defeat induces depressive-like states and microglial activation without involvement of peripheral macrophages. J. Neuroinflammation.

[27] Tashima R (2016). Bone marrow-derived cells in the population of spinal microglia after peripheral nerve injury. Sci. Rep..

[28] Ajami B, Bennett JL, Krieger C, Tetzlaff W, Rossi FM (2007). Local self-renewal can sustain CNS microglia maintenance and function throughout adult life. Nat. Neurosci..

[29] Willis EF (2020). Repopulating microglia promote brain repair in an IL-6-dependent manner. Cell.

[30] Guimaraes RM (2019). Frontline science: blood-circulating leukocytes fail to infiltrate the spinal cord parenchyma after spared nerve injury. J. Leukoc. Biol..

[31] Keren-Shaul H (2017). A unique microglia type associated with restricting development of Alzheimer’s disease. Cell.

[32] Parkhurst CN (2013). Microglia promote learning-dependent synapse formation through brain-derived neurotrophic factor. Cell.

[33] Macosko EZ (2015). Highly parallel genome-wide expression profiling of individual cells using nanoliter droplets. Cell.

[34] Masuda T (2020). Novel Hexb-based tools for studying microglia in the CNS. Nat. Immunol..

[35] Saederup N (2010). Selective chemokine receptor usage by central nervous system myeloid cells in CCR2-red fluorescent protein knock-in mice. PLoS ONE.

[36] Tirosh I (2016). Dissecting the multicellular ecosystem of metastatic melanoma by single-cell RNA-seq. Science.

[37] Scialdone A (2016). Resolving early mesoderm diversification through single-cell expression profiling. Nature.

[38] Stuart T (2019). Comprehensive integration of single-cell data. Cell.

[39] Kohno K (2018). Temporal kinetics of microgliosis in the spinal dorsal horn after peripheral nerve injury in rodents. Biol. Pharm. Bull..

[40] Dang CV (2012). MYC on the path to cancer. Cell.

[41] Haimon Z (2018). Re-evaluating microglia expression profiles using RiboTag and cell isolation strategies. Nat. Immunol..

[42] de Alboran IM (2001). Analysis of C-MYC function in normal cells via conditional gene-targeted mutation. Immunity.

[43] Love MI, Huber W, Anders S (2014). Moderated estimation of fold change and dispersion for RNA-seq data with DESeq2. Genome Biol..

[44] Voet S (2018). A20 critically controls microglia activation and inhibits inflammasome-dependent neuroinflammation. Nat. Commun..

[45] Tavares RM (2010). The ubiquitin modifying enzyme A20 restricts B cell survival and prevents autoimmunity. Immunity.

[46] Kalous, J., Jansova, D. & Susor, A. Role of cyclin-dependent kinase 1 in translational regulation in the M-phase. *Cells*, **9**, 1568. 10.3390/cells9071568 (2020). 10.3390/cells9071568 PMC7408968 32605021

[47] Garcia-Gutierrez L (2019). Myc stimulates cell cycle progression through the activation of Cdk1 and phosphorylation of p27. Sci. Rep..

[48] Wang X (2011). Phosphorylation regulates c-Myc’s oncogenic activity in the mammary gland. Cancer Res.

[49] Okubo M (2016). Macrophage-colony stimulating factor derived from injured primary afferent induces proliferation of spinal microglia and neuropathic pain in rats. PLoS ONE.

[50] Malynn BA, Ma A (2019). A20: A multifunctional tool for regulating immunity and preventing disease. Cell Immunol..

[51] Das T, Chen Z, Hendriks RW, Kool M (2018). A20/Tumor necrosis factor alpha-induced protein 3 in immune cells controls development of autoinflammation and autoimmunity: lessons from mouse models. Front. Immunol..

[52] Chen S (2015). Up-regulated A20 promotes proliferation, regulates cell cycle progression and induces chemotherapy resistance of acute lymphoblastic leukemia cells. Leuk. Res..

[53] Sanz E (2009). Cell-type-specific isolation of ribosome-associated mRNA from complex tissues. Proc. Natl Acad. Sci. USA.

[54] Greten FR (2004). IKKbeta links inflammation and tumorigenesis in a mouse model of colitis-associated cancer. Cell.

[55] Martin, E., El-Behi, M., Fontaine, B. & Delarasse, C. Analysis of microglia and monocyte-derived macrophages from the central nervous system by flow cytometry. *J. Vis. Exp*. **22**, 55781.10.3791/55781 (2017). 10.3791/55781 PMC5608497 28671658

[56] Bohlen CJ (2017). Diverse requirements for microglial survival, specification, and function revealed by defined-medium cultures. Neuron.

[57] Zheng GX (2017). Massively parallel digital transcriptional profiling of single cells. Nat. Commun..

[58] RStudio. *R Studio: Integrated Development for R. RStudio*, http://www.rstudio.com (2020).

[59] Butler A, Hoffman P, Smibert P, Papalexi E, Satija R (2018). Integrating single-cell transcriptomic data across different conditions, technologies, and species. Nat. Biotechnol..

