# Supplementary material for: Distinct phases of adult microglia proliferation: a Myc-mediated early phase and a Tnfaip3-mediated late phase
Source: Cell Discov. 2022 Apr 12;8:34. doi: 10.1038/s41421-022-00377-3 (PMC9001707; doi:10.1038/s41421-022-00377-3)
Supplement: Supplementary file 1 — Supplementary Information [file 41421_2022_377_MOESM1_ESM.pdf]

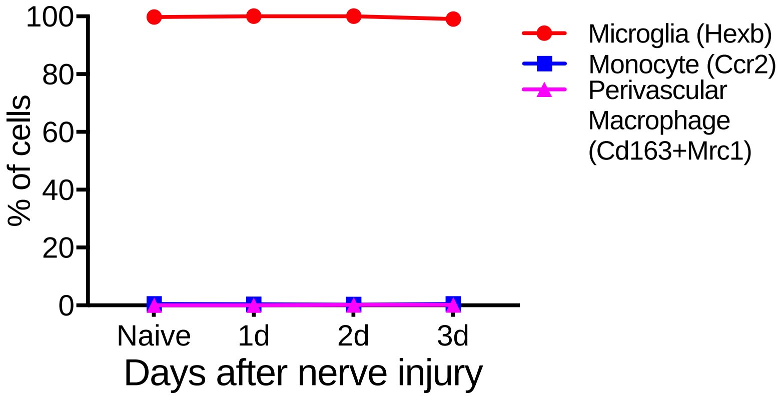

Supplementary Fig. S1. Virtually all *Cx3cr1*-YFP (+) cells in mouse lumbar spinal cord are microglia.

scRNA-Seq analysis of microglia and monocyte markers on *Cx3cr1*-YFP (+) cells in mouse lumbar spinal cord. More than 99% of *Cx3cr1*-YFP (+) cells from mouse lumbar cord are *Hexb*-expressing microglia, with minimal *Ccr2*-expressing monocyte, or *Cd163* and *Mrc1*-expressing perivascular macrophages in naïve animals or animals after nerve injury.

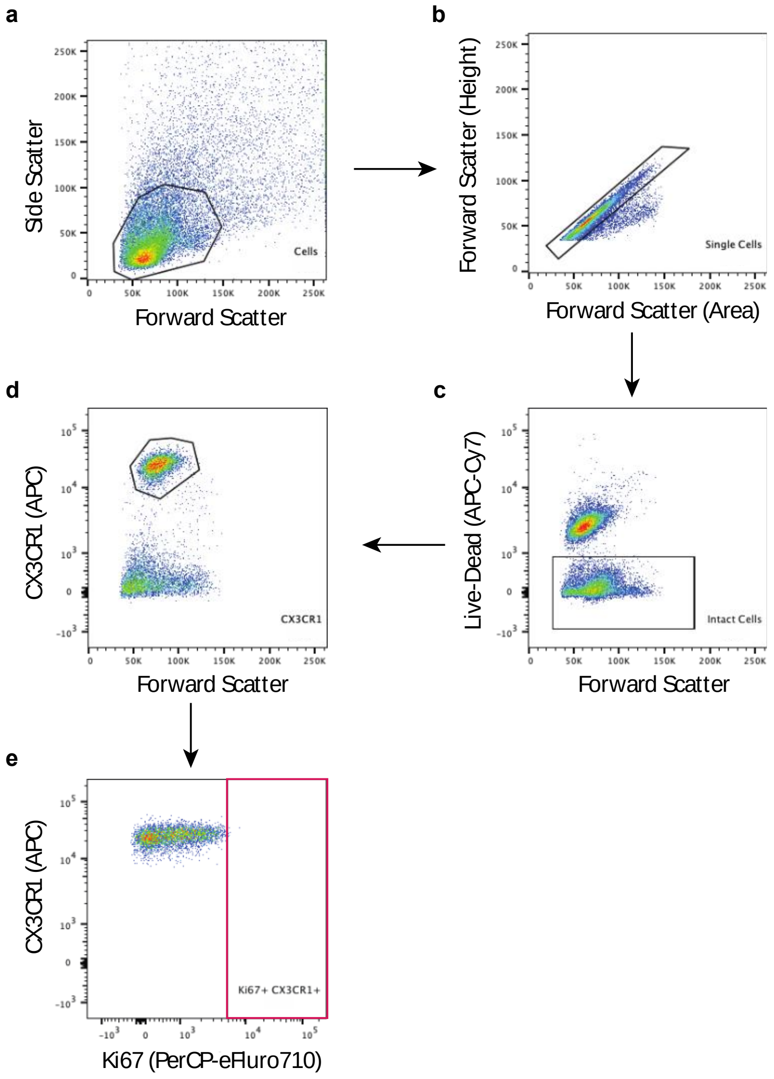

Supplementary Fig. S2. Gating strategy to analyze microglia by flow cytometry.

Representative flow cytometry scatter plots. Forward versus side scatter gating was used to identify cells of interest based on size and granularity (**a**), and the single cells were selected by plotting forward scatter height over forward scatter area (**b**). Intact cells were then selected based on non-reactive staining with Live/Dead cell viability dye (**c**), and microglia were identified as the CX3CR1(+) population (**d**). Ki67 distinguished proliferating microglia, and the Ki67 gate was determined using "fluorescence minus one" method with the appropriate isotype PerCP-eFluo710 fluorophore (**e**).

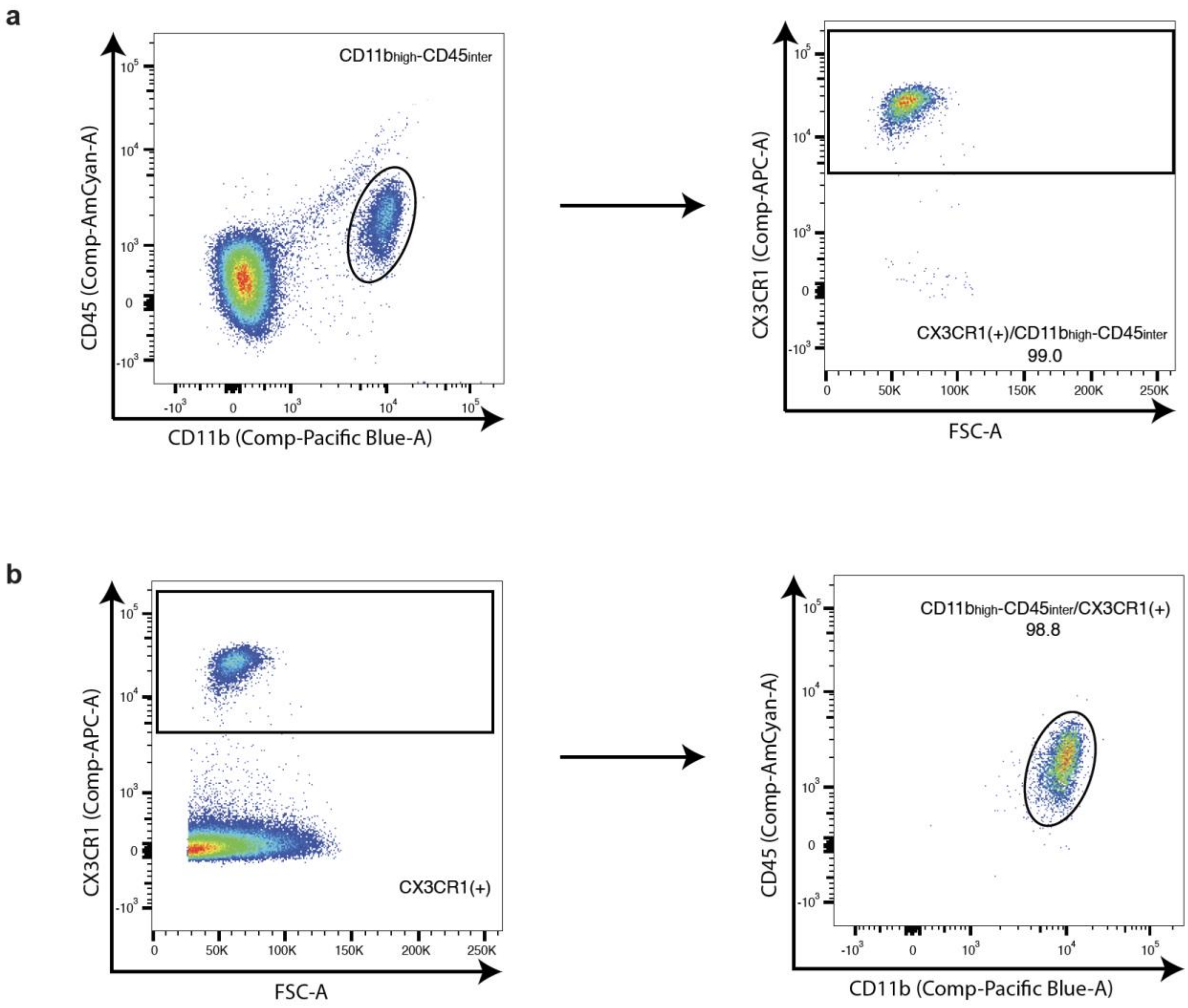

Supplementary Fig. S3. Characterization of spinal cord microglia by flow cytometry.

Representative flow cytometry scatter plots. **a.** 99% of CD11b^{high}-CD45^{inter} cells in mouse spinal cord were CX3CR1 (+). **b.** 98.8% of CX3CR1 (+) cells in mouse spinal cord were CD11b^{high}-CD45^{inter}.

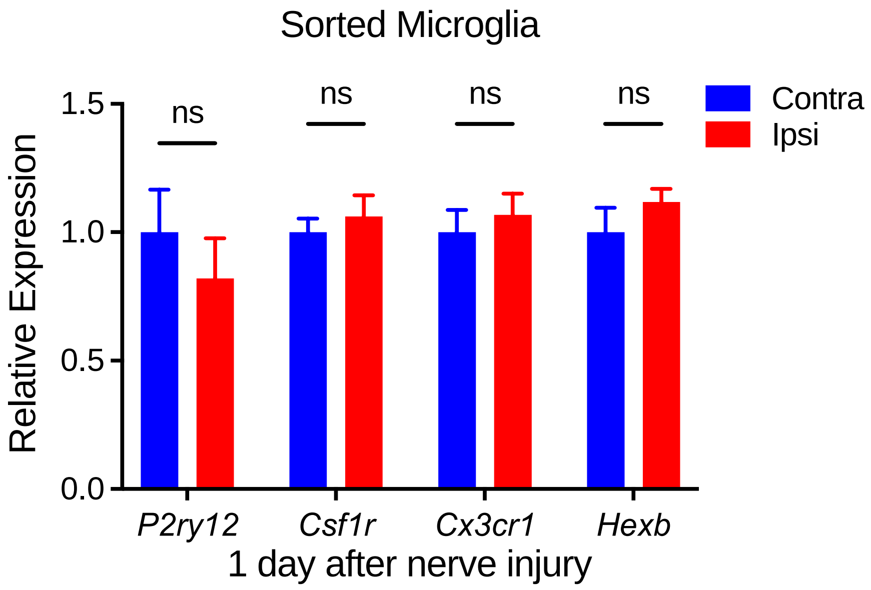

Supplementary Fig. S4. Unchanged expression of microglia marker genes 1 day after nerve injury.

qRT-PCR of microglia sorted from lumbar spinal cord. The expression of microglia marker genes (*P2ry12*, *Csf1r*, *Cx3cr1*, and *Hexb*) was unchanged 1 day after nerve injury.

Two-way ANOVA with Sidak's multiple comparisons test, n=4, mean ± s.e.m., and ns = not significant.

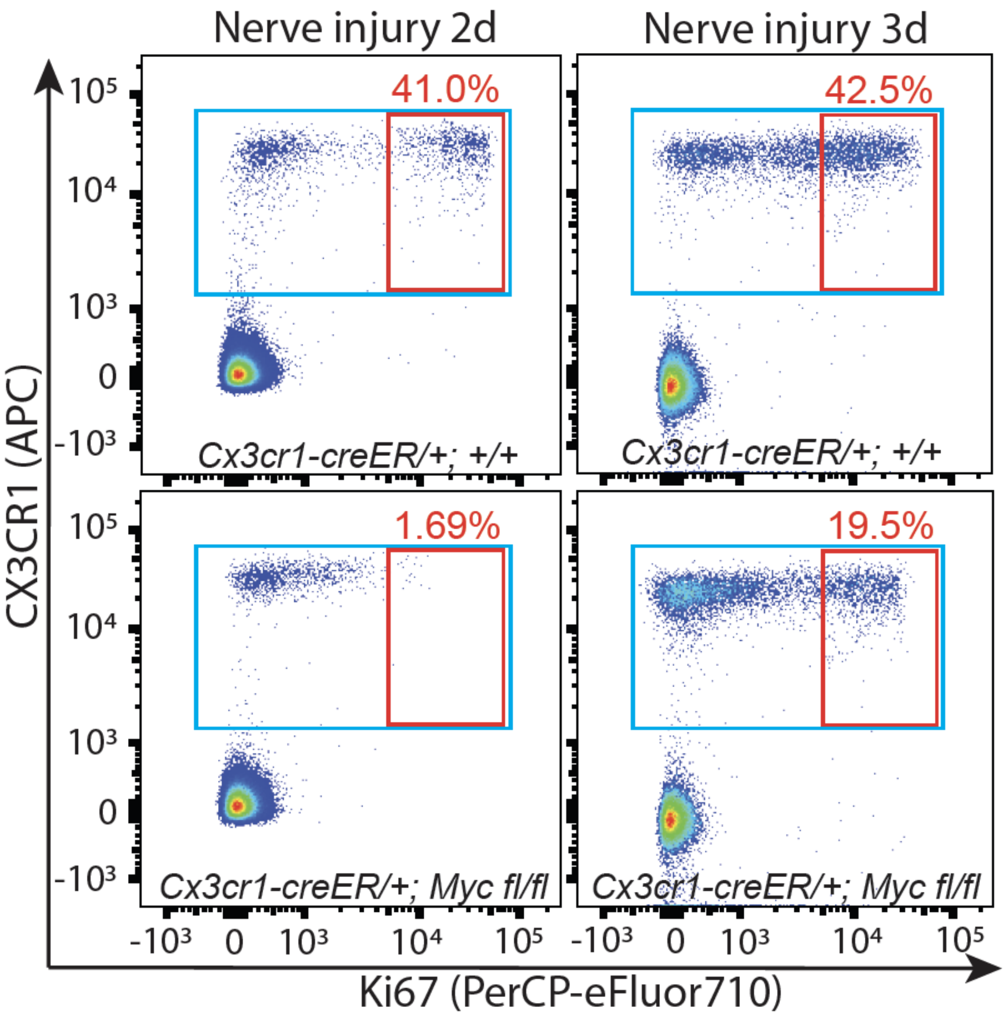

Supplementary Fig. S5. The early phase of microglia proliferation is *Myc* dependent. Representative flow cytometry scatter plots. *Myc* deletion from adult microglia prevented the early phase of lumbar cord microglia proliferation, which occurred 2 days after sciatic nerve injury. However, the late phase of microglia proliferation on day 3 after nerve injury remained in microglia with *Myc* deletion.

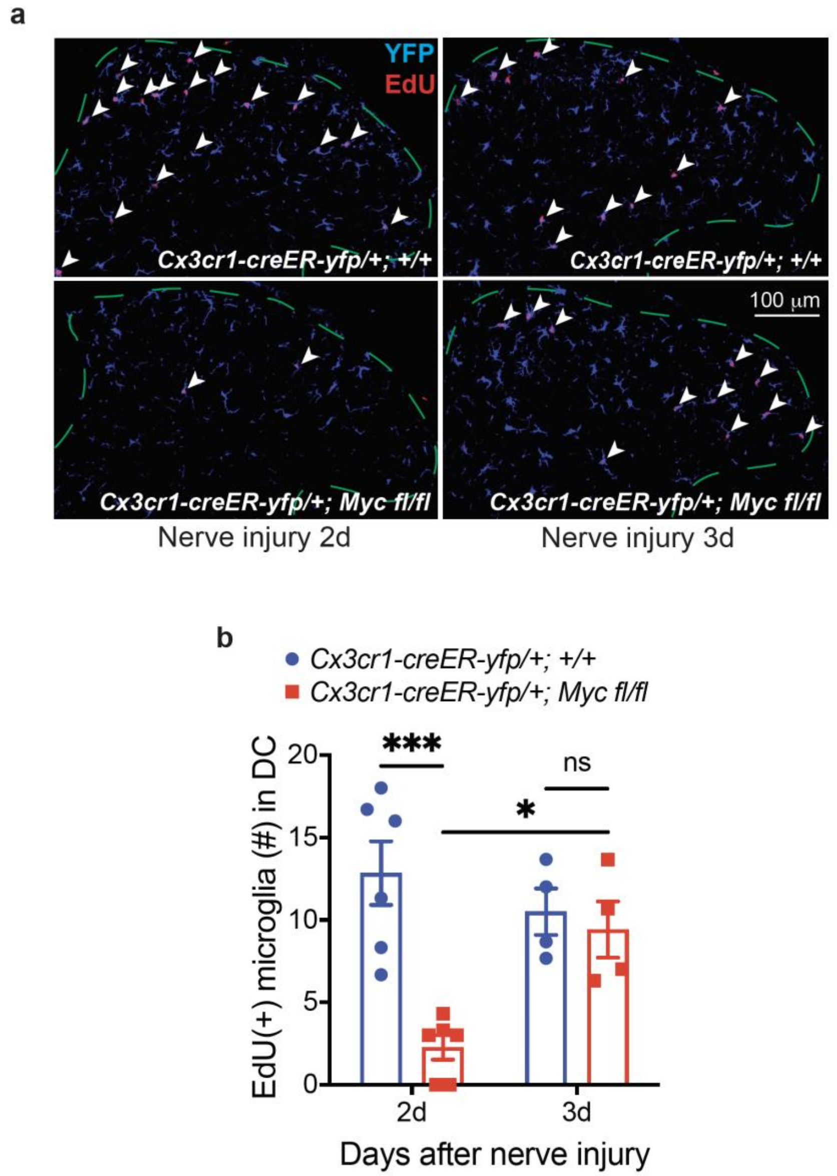

Supplementary Fig. S6. *Myc*-dependent early phase microglia proliferation

Representative EdU IHC staining (**a**) and the associated quantification (**b**) of lumbar dorsal cord microglia after nerve injury. Animals with *Myc* deletion from adult microglia had significantly less EdU (+) proliferating microglia in lumbar dorsal cord (DC) 2 days, but not 3 days, after nerve injury. The arrowheads point to the EdU (+) microglia, and the dashed lines outline the dorsal lumbar cord. Microglia are identified by YFP staining. Two-way ANOVA with Sidak's multiple comparisons test, n=4-6, mean ± s.e.m., * $p<0.05$, and *** $p<0.001$.

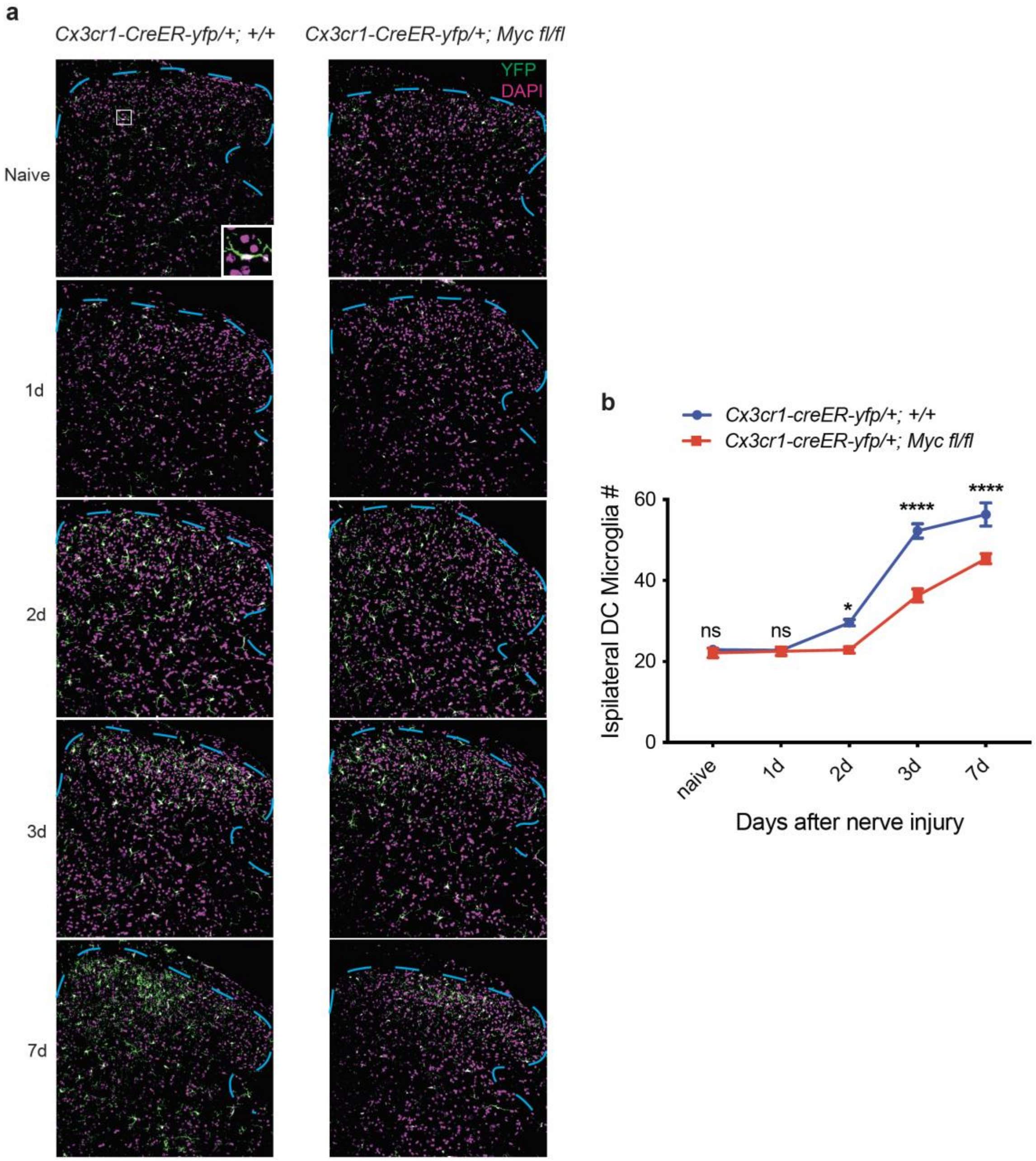

Supplementary Fig. S7. Nerve injury-induced microgliosis is reduced in *Myc* cKO mice. Representative IHC images (**a**) and the associated quantification (**b**) of dorsal cord (DC) microglia at different time points after nerve injury. Although deleting *Myc* cKO mice had normal number of microglia in lumbar dorsal cord in naïve animals, microgliosis induced by nerve injury was significantly less in *Myc* cKO mice. Two-way ANOVA with Sidak's multiple comparisons test, n=3-5, mean ± s.e.m., * $p<0.05$, **** $p<0.0001$, and ns = not significant.

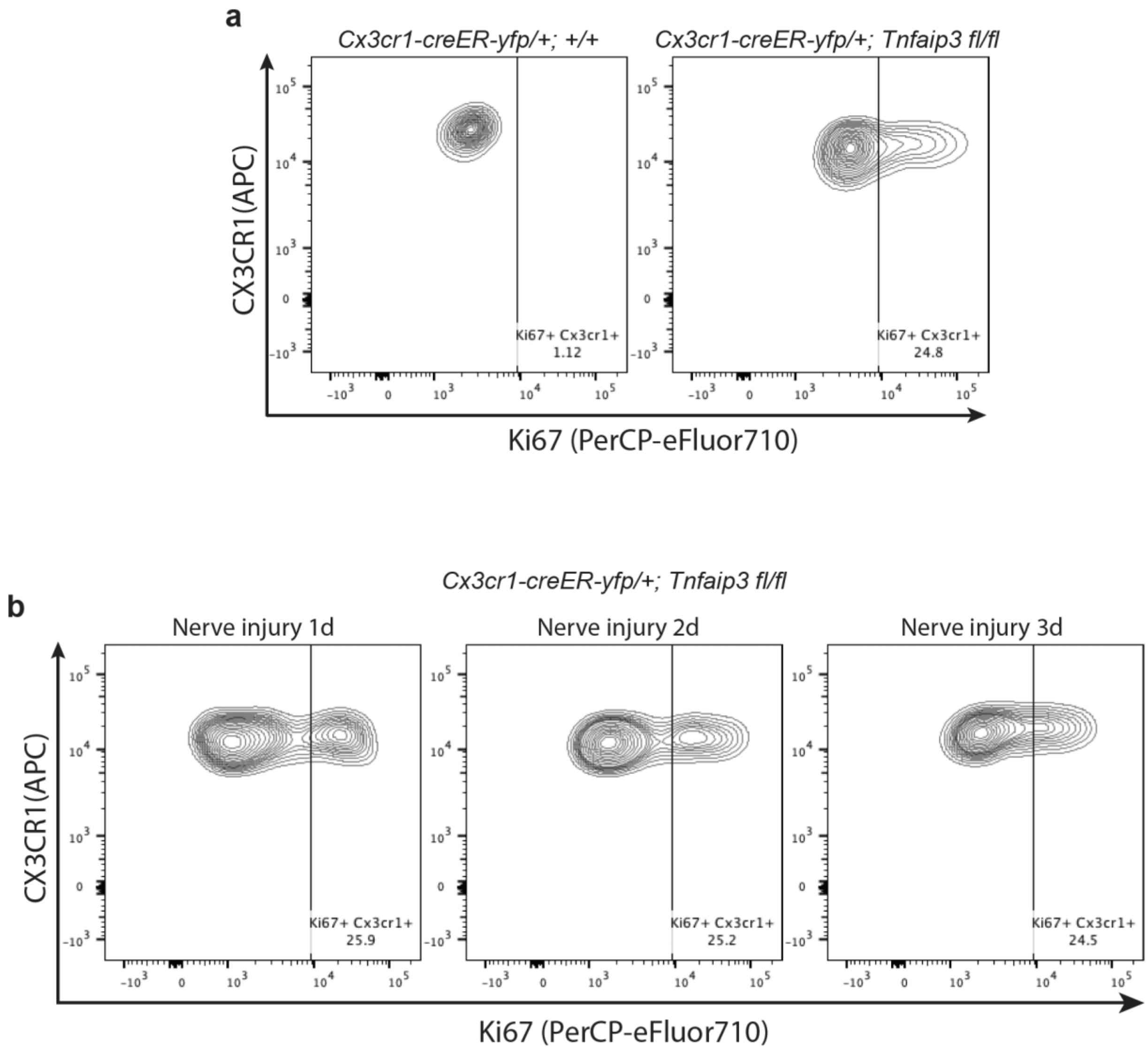

Supplementary Fig. S8. A20 inhibits microglia proliferation.

Representative flow cytometry scatter plots. **a.** *Tnfaip3* deletion from adult microglia resulted in increased microglia proliferation in the absence of nerve injury. **b.** Nerve injury had no further effect on microglia proliferation in microglia lacking *Tnfaip3*.

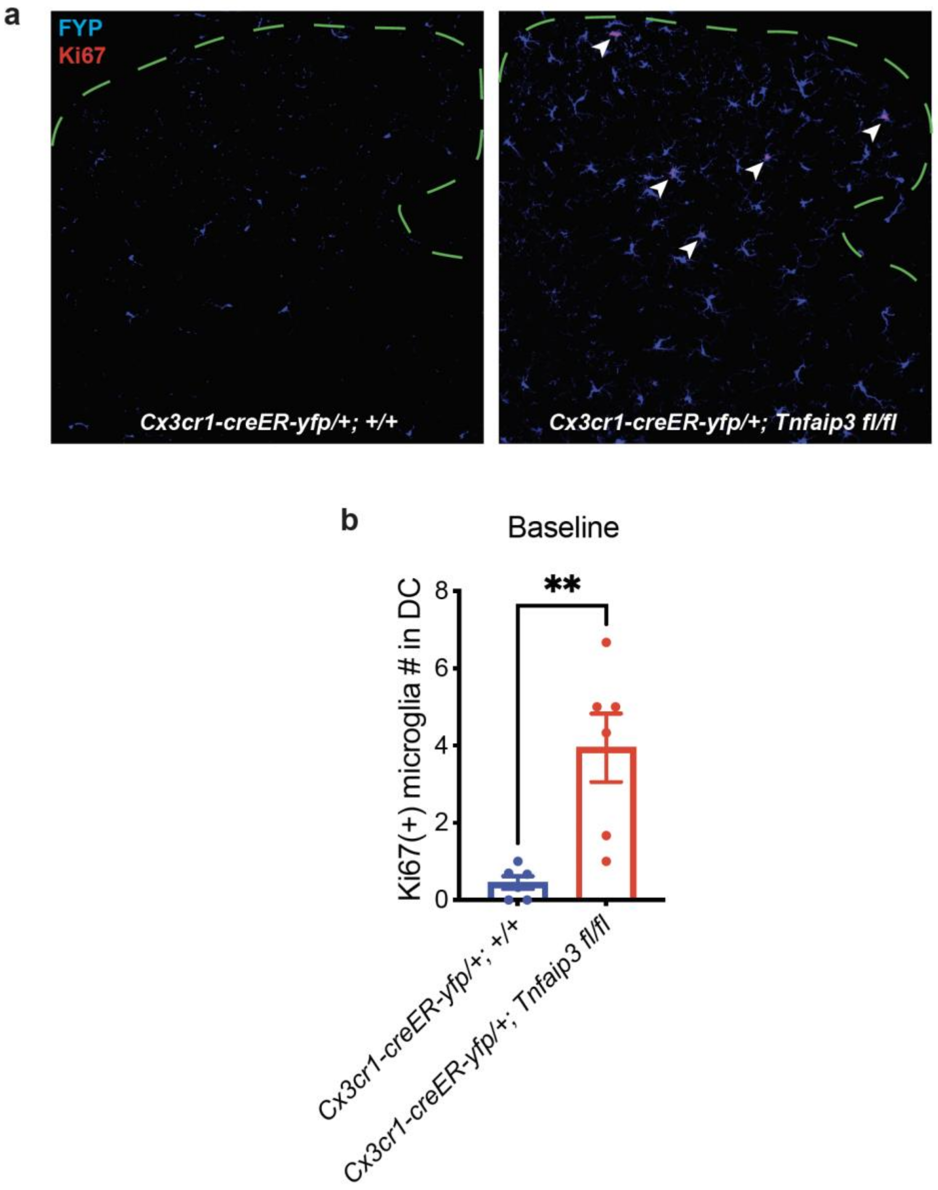

Supplementary Fig. S9. Increased microglia proliferation in dorsal cord of naïve A20 cKO mice.

Representative IHC images (**a**) and the associated quantification (**b**) showing that A20 cKO mice had increased dorsal cord (DC) microglia proliferation in the absence of nerve injury. Unpaired two-tailed t-test, n=4-6, mean ± s.e.m., ** p<0.01.

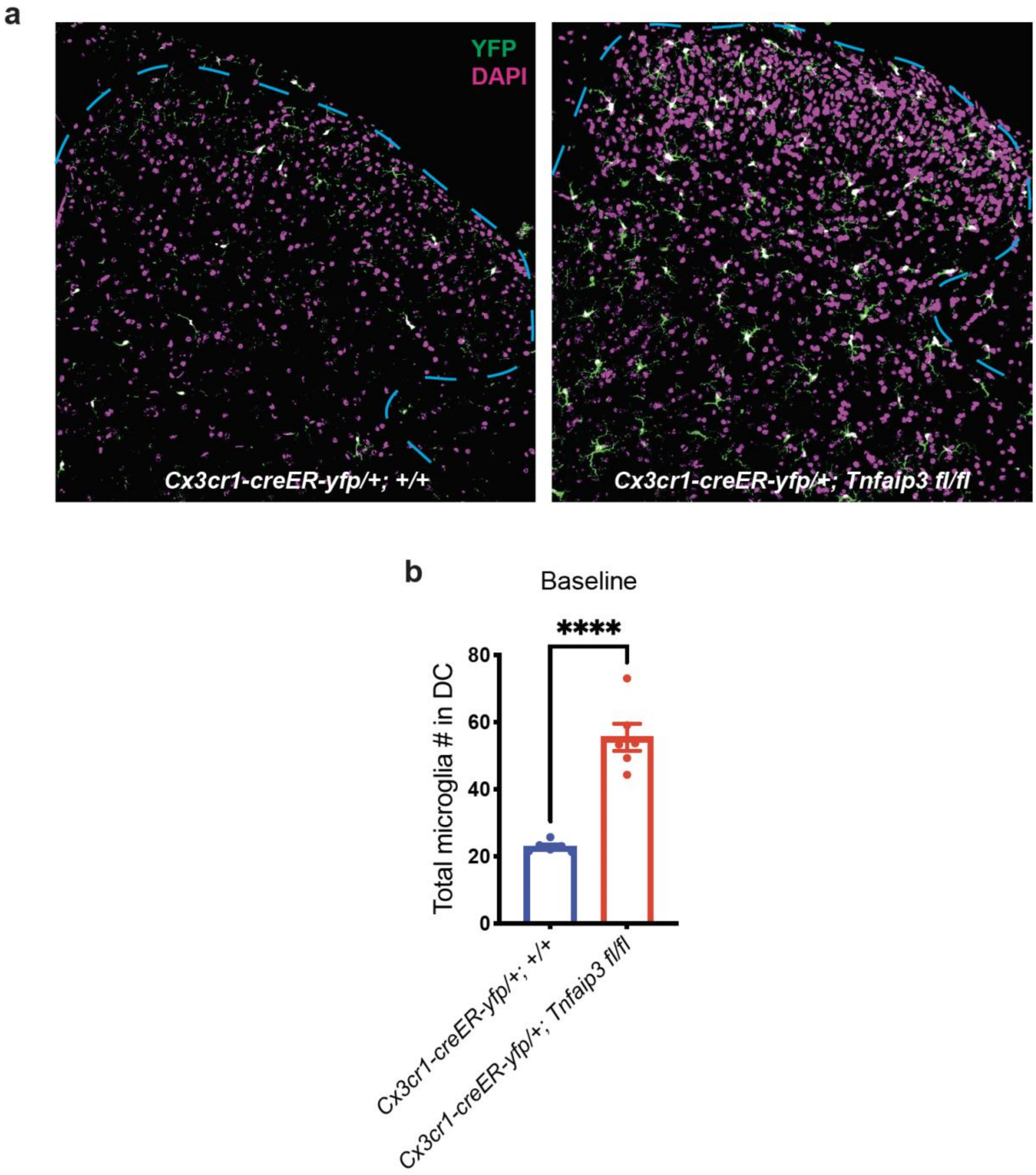

Supplementary Fig. S10. Increased microglia number in dorsal cord of A20 cKO mice. Representative IHC images (**a**) and the associated quantification (**b**) showing that A20 cKO mice had increased dorsal cord (DC) microglia in the absence of nerve injury. Unpaired two-tailed t-test, n=4-6, mean ± s.e.m., **** p<0.0001.

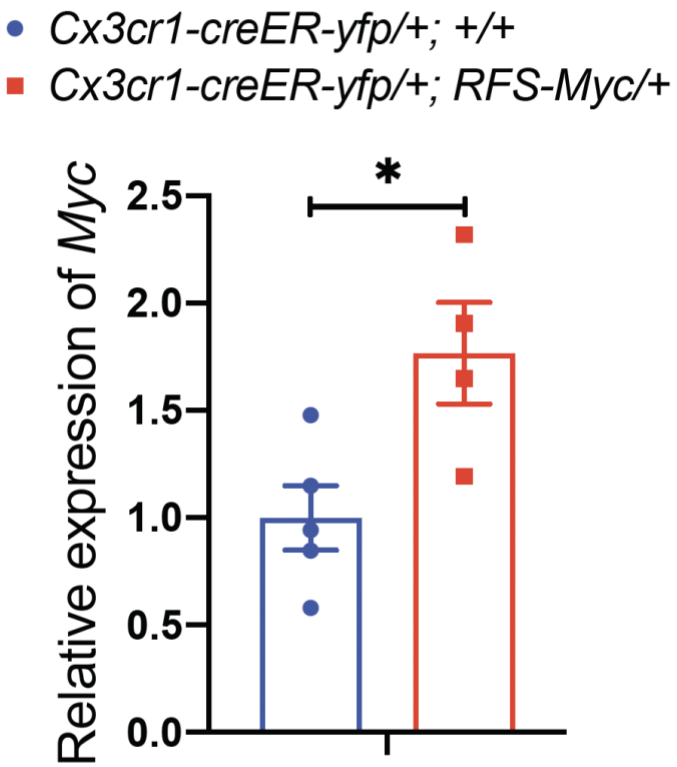

Supplementary Fig. S11. *Myc* overexpression in microglia of *Cx3cr1-creER-yfp/+; RFS-Myc/+* mice.

qRT-PCR of sorted spinal cord microglia. *Myc* was overexpressed in the microglia of adult naive *Cx3cr -creER-yfp/+; RFS-Myc/+* animal after tamoxifen treatment. Unpaired two-tailed t test, n=4-5, mean ± s.e.m., * $p<0.05$.

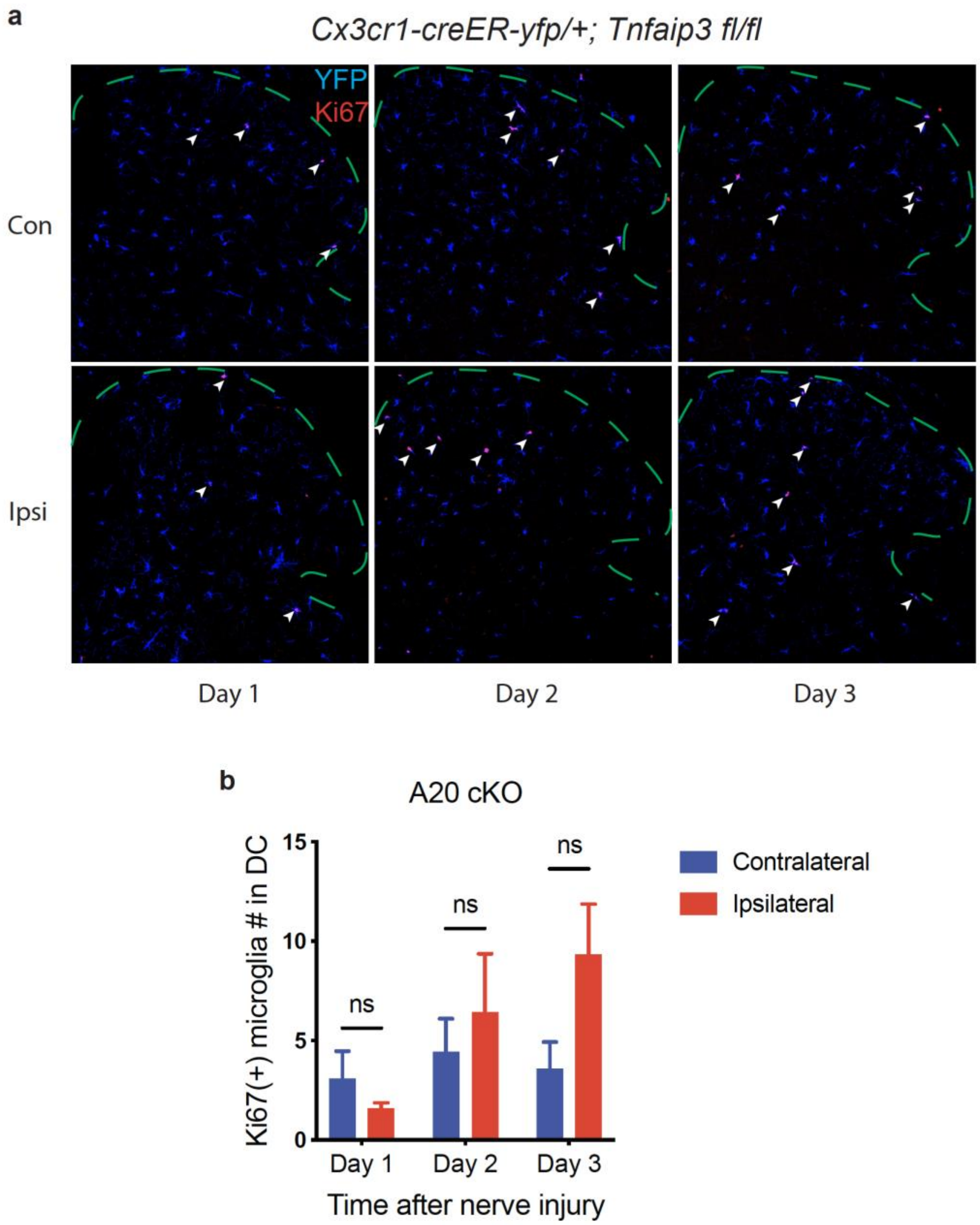

Supplementary Fig. S12. A20 cKO mice did not have early phase dorsal cord microglia proliferation 2 days after nerve injury.

Representative IHC images (**a**) and the associated quantification (**b**) showing that A20 cKO mice did not have increased dorsal cord (DC) microglia proliferation after nerve injury. Two-way ANOVA with Sidak's multiple comparisons test, n=4, mean ± s.e.m., ns = not significant.

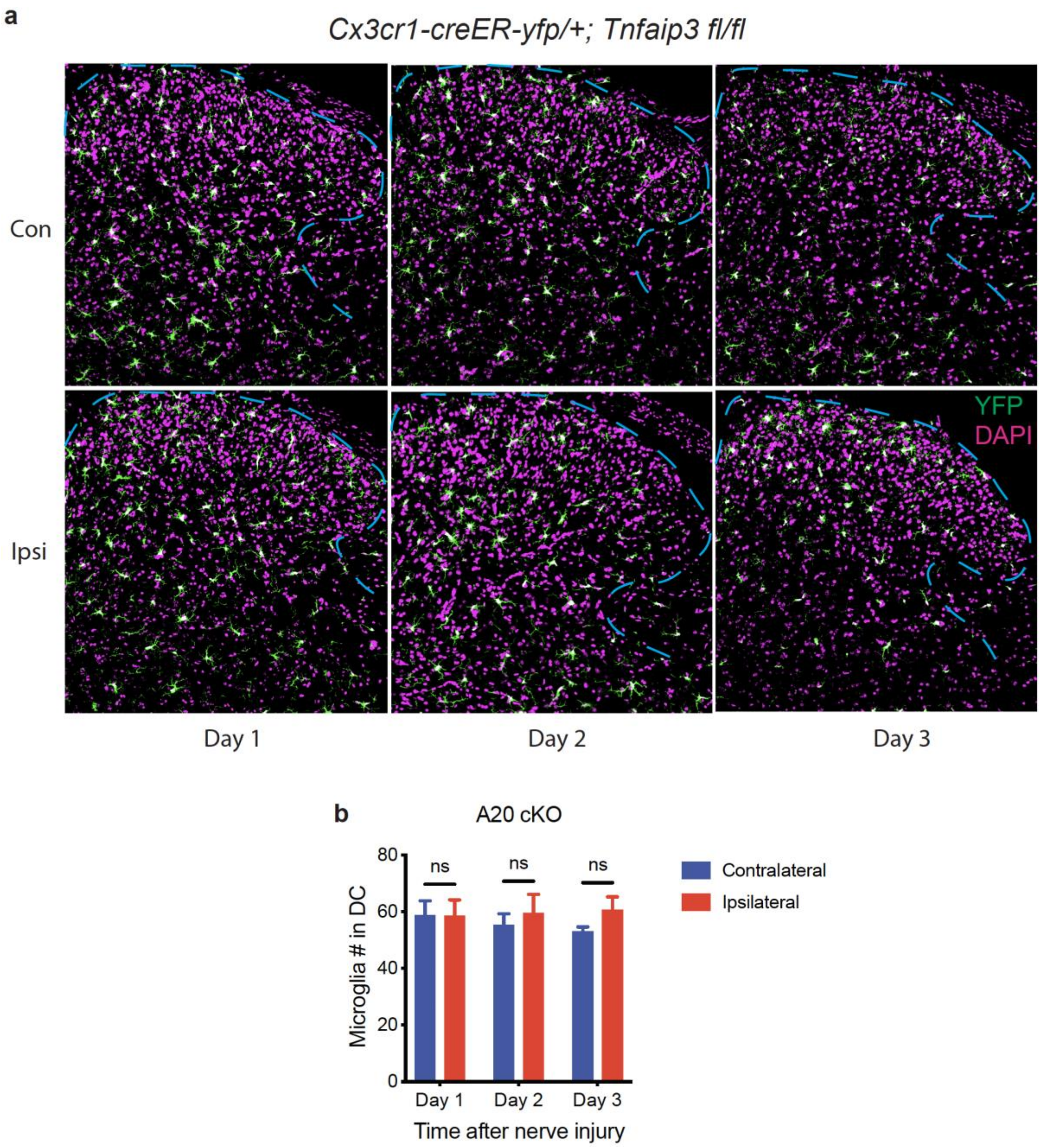

Supplementary Fig. S13. Nerve injury did not induce further microgliosis in the dorsal cord of A20 cKO mice.

Representative IHC images (**a**) and the associated quantification (**b**) showing that A20 cKO mice did not have increased dorsal cord (DC) microglia number after nerve injury. Two-way ANOVA with Sidak's multiple comparisons test, n=4, mean ± s.e.m., ns = not significant.

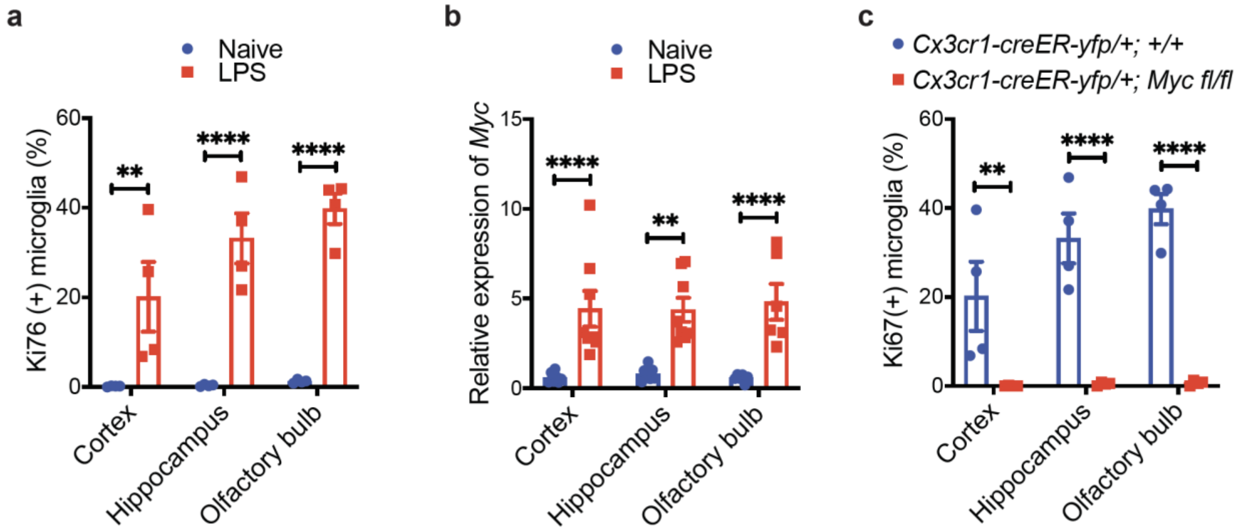

Supplementary Fig. S14. LPS-induced microglia proliferation is *Myc* dependent across multiple brain regions.

a. Quantification of flow cytometry. A single intraperitoneal injection of LPS induced microglia proliferation in cortex, hippocampus, and olfactory bulb 2 days after the treatment.

b. qRT-PCR of sorted microglia. Intraperitoneal injection of LPS upregulated *Myc* in microglia of cortex, hippocampus, and olfactory bulb 4 hours after the treatment.

c. Quantification of flow cytometry. *Myc* deletion in adult microglia prevented LPS-induced microglia proliferation in cortex, hippocampus, or olfactory bulb.

Two-way ANOVA with Sidak's multiple comparisons test, n=4-6, mean ± s.e.m., ** $p<0.01$, and **** $p<0.0001$.

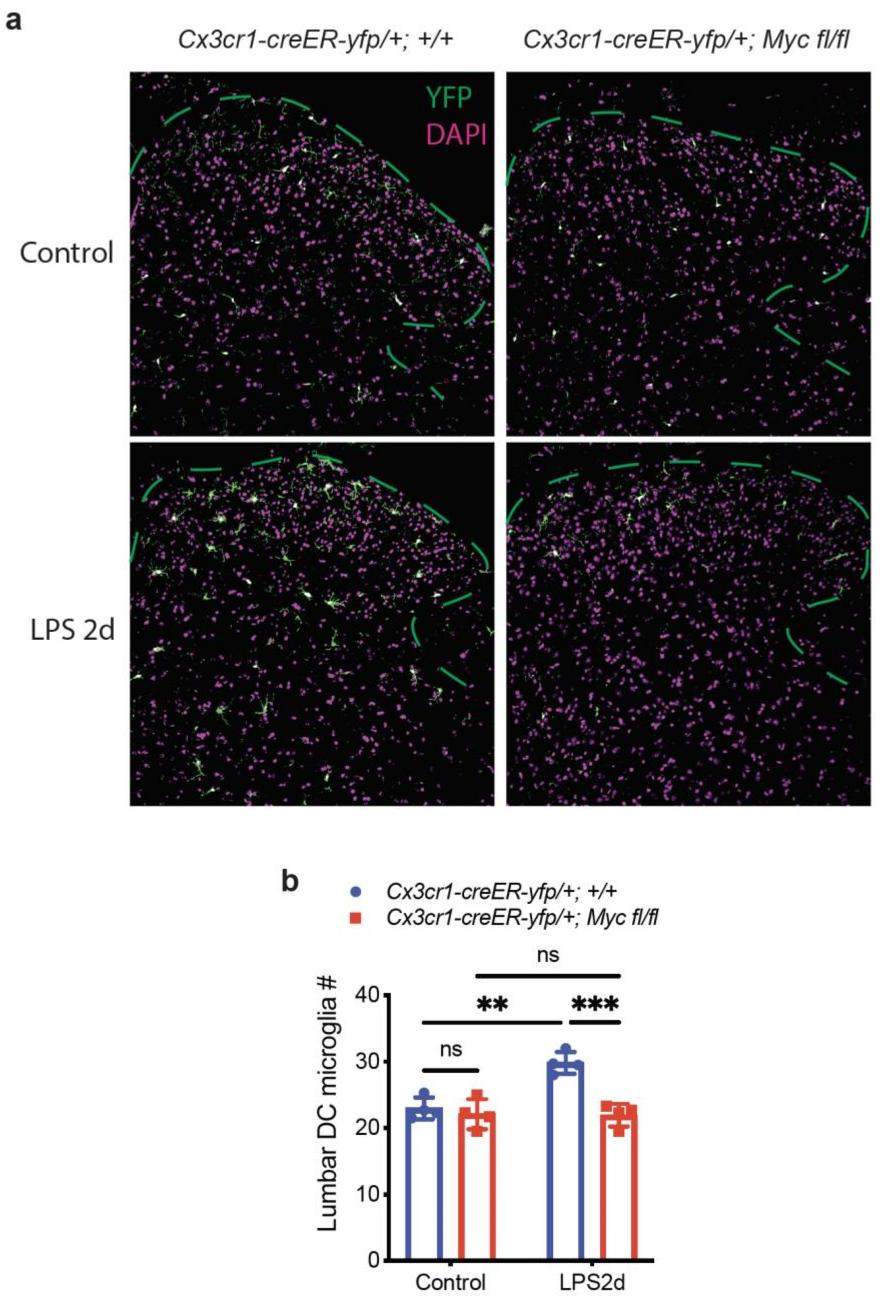

Supplementary Fig. S15. LPS-induced microgliosis is prevented in *Myc* cKO mice.

Representative images (**a**) and the associated quantification (**b**) showing that Deleting *Myc* from adult microglia prevented lumbar dorsal cord (DC) microgliosis 2 days after LPS. Two-way ANOVA with Sidak's multiple comparisons test, n=4, mean ± s.e.m., ** $p<0.01$, *** $p<0.001$, and ns = not significant.

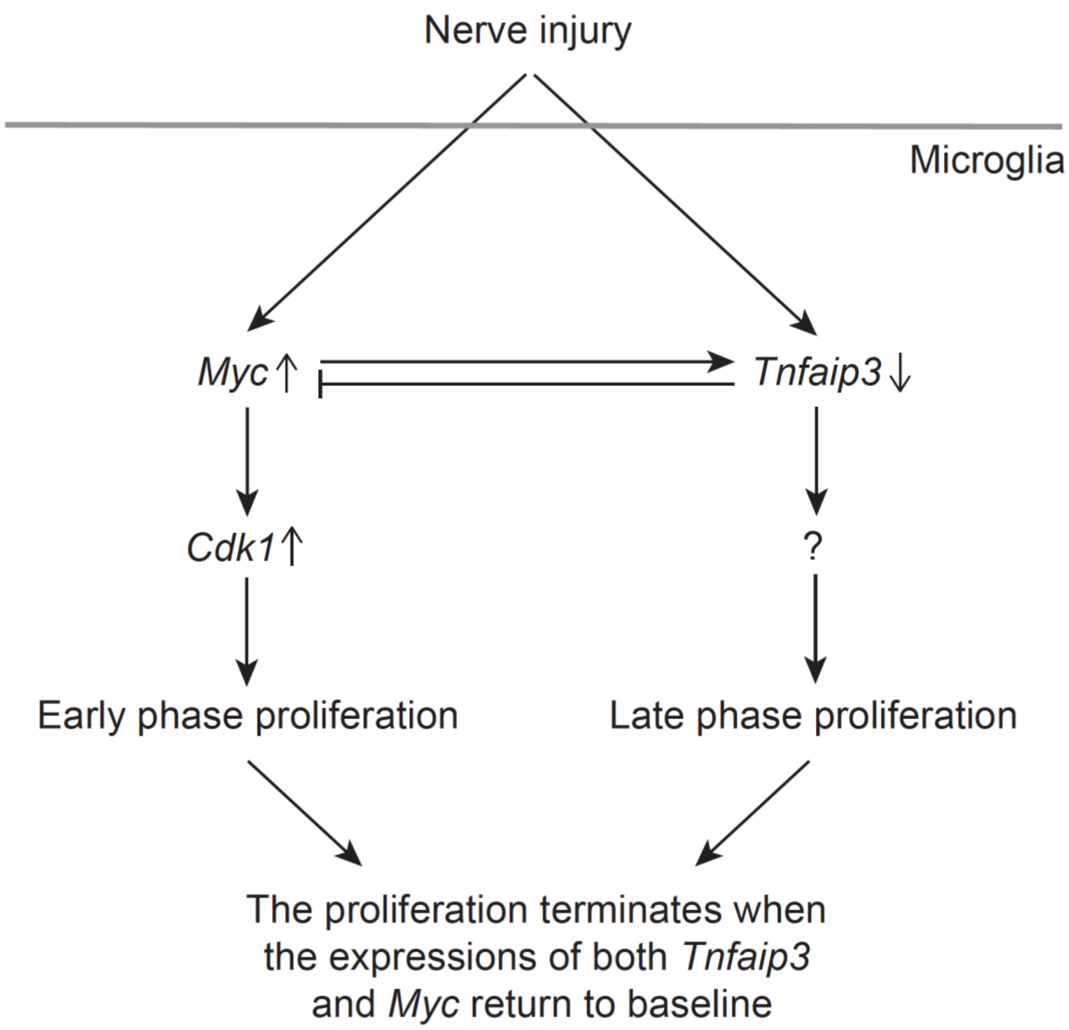

Supplementary Fig. S16. Signaling pathways for microglia proliferation.

Microglia proliferation consists of a *Myc*-dependent early phase, a *Myc*-independent late phase, and a termination phase. Peripheral nerve injury leads to *Myc* upregulation in microglia, which in turns induces *Cdk1* upregulation to mediate early phase microglia proliferation. In parallel, nerve injury also induces *Tnfaip3* downregulation in microglia, which mediates late phase microglia proliferation. There are cross-talks between the early and late phase proliferation pathways, as *Myc* upregulation contributes to *Tnfaip3* downregulation, and conversely, *Tnfaip3* downregulation inhibits nerve injury-induced *Myc* upregulation. Microglia proliferation terminates when both *Tnfaip3* and *Myc* resume their baseline expression.

Supplementary Table S1. List of genes differentially expressed in G2M phase cells, 2d vs 3d, in Figure 2e

Genes downregulated	Genes upregulated
Gm42418	Tgfb1
Hist1h1e	
Ifit3	
Ifi27l2a	
Ifi204	
Ifitm3	
Rpl10-ps3	

Supplementary Table S2. List of genes differentially expressed in S phase cells, 1d vs 2d, in Figure 3a

Genes downregulated	Genes upregulated
Stmn1	Adamts1
	Atf3
	Btg1
	Btg2
	Ccl3
	Ccl4
	Ccl12
	Dusp1
	Dusp6
	Egr1
	Fos
	Fosb
	H3f3b
	Ier2
	Ier5
	Jun
	Junb
	Jund
	Klf2
	Klf4
	Klf6
	Rgs1
	Rgs2
	Rhob
	Sgk1
	Socs3
	Tmx4
	Ubc
	Zfp36
	Zfp36l1

Supplementary Table S3. List of genes differentially expressed in G2M phase cells, A20 cKO naïve vs control 2d, in Figure 4a

Genes downregulated	Genes upregulated
C1qa	Ccl5
C1qb	H2-D1
Ckb	H2-K1
Cst3	Ifitm3
Fcrls	
Gm10076	
Ltc4s	
mt-Nd1	
Olfml3	
Rplp1	
Trem2	
Tyrobp	
